# Discovery and Biosynthesis of the Antibiotic Bicyclomycin in Distantly Related Bacterial Classes

**DOI:** 10.1128/AEM.02828-17

**Published:** 2018-04-16

**Authors:** Natalia M. Vior, Rodney Lacret, Govind Chandra, Siobhán Dorai-Raj, Martin Trick, Andrew W. Truman

**Affiliations:** a Department of Molecular Microbiology, John Innes Centre, Norwich, UK; b Department of Computational and Systems Biology, John Innes Centre, Norwich, UK; University of California, Davis

**Keywords:** Pseudomonas aeruginosa, Streptomyces, antibiotic, biosynthesis, gene transfer, phylogenetic analysis

## Abstract

Bicyclomycin (BCM) is a clinically promising antibiotic that is biosynthesized by Streptomyces cinnamoneus DSM 41675. BCM is structurally characterized by a core cyclo(l-Ile-l-Leu) 2,5-diketopiperazine (DKP) that is extensively oxidized. Here, we identify the BCM biosynthetic gene cluster, which shows that the core of BCM is biosynthesized by a cyclodipeptide synthase, and the oxidative modifications are introduced by five 2-oxoglutarate-dependent dioxygenases and one cytochrome P450 monooxygenase. The discovery of the gene cluster enabled the identification of BCM pathways encoded by the genomes of hundreds of Pseudomonas aeruginosa isolates distributed globally, and heterologous expression of the pathway from P. aeruginosa SCV20265 demonstrated that the product is chemically identical to BCM produced by S. cinnamoneus. Overall, putative BCM gene clusters have been found in at least seven genera spanning Actinobacteria and Proteobacteria (Alphaproteobacteria, Betaproteobacteria, and Gammaproteobacteria). This represents a rare example of horizontal gene transfer of an intact biosynthetic gene cluster across such distantly related bacteria, and we show that these gene clusters are almost always associated with mobile genetic elements.

**IMPORTANCE** Bicyclomycin is the only natural product antibiotic that selectively inhibits the transcription termination factor Rho. This mechanism of action, combined with its proven biological safety and its activity against clinically relevant Gram-negative bacterial pathogens, makes it a very promising antibiotic candidate. Here, we report the identification of the bicyclomycin biosynthetic gene cluster in the known bicyclomycin-producing organism Streptomyces cinnamoneus, which will enable the engineered production of new bicyclomycin derivatives. The identification of this gene cluster also led to the discovery of hundreds of bicyclomycin pathways encoded in highly diverse bacteria, including in the opportunistic pathogen Pseudomonas aeruginosa. This wide distribution of a complex biosynthetic pathway is very unusual and provides an insight into how a pathway for an antibiotic can be transferred between diverse bacteria.

## INTRODUCTION

**FIG 1:**
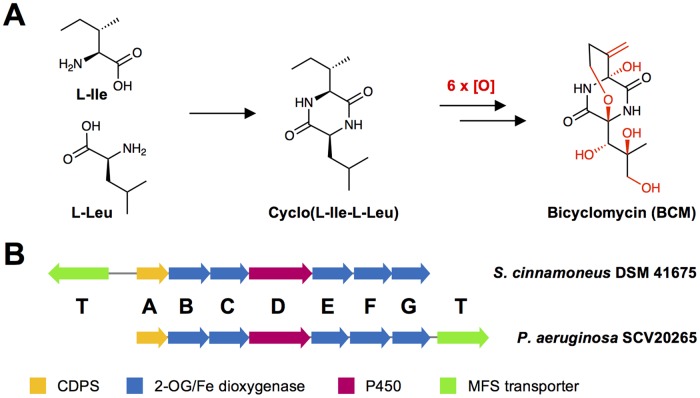
Bicyclomycin biosynthesis. (A) Simplified schematic of BCM biosynthesis. (B) *bcm* gene clusters identified in S. cinnamoneus and P. aeruginosa.

Bicyclomycin (BCM) is a broad-spectrum antibiotic active against Gram-negative bacteria that was first isolated in 1972 from Streptomyces cinnamoneus (originally named Streptomyces sapporoensis) (1) and is also produced by two other Streptomyces species (2, 3). BCM (also known as bicozamycin) is one of the most complex members of the 2,5-diketopiperazine (DKP) family of molecules, which are cyclic dipeptides generated by the head-to-tail condensation of two α-amino acids (4). The core DKP of BCM, cyclo(l-Ile-l-Leu) (cIL), is modified with a characteristic second cycle that forms a [4.2.2] bicyclic unit, an exomethylene group, and multiple hydroxylations (5) (Fig. 1A). BCM is a selective inhibitor of the transcription termination factor Rho (6), which is an essential protein in many bacteria (7, 8) and has been used to treat traveler's diarrhea (9), as well as in veterinary medicine to treat calves, pigs, and fish (7).

BCM is the only natural product known to target Rho, which together with its proven safety in mammals and its activity against clinically relevant Gram-negative bacterial pathogens, like Acinetobacter baumannii and Klebsiella pneumoniae, makes it a very attractive antibiotic (7, 10). This promise is enhanced by the recent discovery that a combination of BCM with bacteriostatic concentrations of antibiotics targeting protein synthesis leads to a rapid bactericidal synergy (10). Furthermore, structure-activity relationship studies show that BCM potency can be improved through modification of its exomethylene group (11, 12).

In contrast with the extensive knowledge on BCM's mechanism of action (6, 7), very little was known about the biosynthesis of this antibiotic. Feeding experiments previously showed that the DKP scaffold derives from l-leucine and l-isoleucine, as well as the likely involvement of a cytochrome P450 monooxygenase in one of the oxidative steps that convert cIL into BCM (13) (Fig. 1A). To understand BCM biosynthesis, we identified the biosynthetic gene cluster for BCM in S. cinnamoneus DSM 41675, which showed that the DKP core is produced by a cyclodipeptide synthase (CDPS) (Fig. 1B). This discovery enabled the identification of homologous clusters in several other species, including hundreds of isolates of Pseudomonas aeruginosa, an opportunistic pathogen that causes serious hospital-acquired infections. We prove that the P. aeruginosa bcm gene cluster is functional and that its product is identical to BCM from Streptomyces; therefore, it represents a viable alternative platform for BCM production. This is a rare example of an almost identical biosynthetic gene cluster in Gram-negative and Gram-positive bacteria. An analysis of the phylogeny and genomic context of *bcm* gene clusters provides an insight into its likely dispersion through horizontal gene transfer (HGT) and implies that the *bcm* gene cluster may have undergone a partial genetic rearrangement between Gram-positive and Gram-negative bacteria.

## RESULTS AND DISCUSSION

### Genome sequencing and identification of the BCM gene cluster in S. cinnamoneus.

The genome sequence of a known BCM producer, S. cinnamoneus DSM 41675, was obtained using a combination of Oxford Nanopore MinION and Illumina MiSeq technologies. Illumina MiSeq provided accurate nucleotide-level read data, but an Illumina-only assembly was distributed across 415 contigs, in part due to the difficulties in assembling short-read data of highly repetitive sequences from large modular polyketide synthase (PKS) and nonribosomal peptide synthetase (NRPS) genes (14), which were found at the start or end of multiple contigs. Therefore, we also sequenced the genome using Oxford Nanopore MinION technology, which is capable of achieving read lengths of over 150 kb (15). The Nanopore output enabled a much better assembly of the genome over 4 contigs, although at a much lower accuracy at the nucleotide level. Using the raw read data from both sequence runs, we obtained a hybrid assembly composed of a 6.46-Mb contig containing almost all of the chromosome, as well as a smaller 199-kb contig (see Table S1 in the supplemental material). antiSMASH analysis (16) of this assembly revealed that the 199-kb contig is likely to form part of the chromosome, as the termini of this contig and the 6.46-Mb contig encode different regions of an enduracidin-like gene cluster. In total, these two contigs yield an almost-contiguous 6.66-Mb S. cinnamoneus genome sequence.

Published feeding experiments indicate that BCM is a DKP derived from l-leucine and l-isoleucine and that a cytochrome P450 is likely to be involved in the pathway (13). Furthermore, a number of additional oxidative reactions are needed to form the final molecule (Fig. 1A). DKPs are produced naturally by either bimodular NRPSs (17, 18) or by CDPSs (19–21), so we expected the biosynthetic gene cluster for BCM to encode either of these enzymatic systems plus six to seven oxidative enzymes. Analysis of the S. cinnamoneus genome sequence with antiSMASH 3.0.5 (16) indicated that there were no suitable NRPS pathways but also no identifiable CDPS pathways. We therefore assessed the genomic regions surrounding every P450 gene in the genome, which revealed the presence of a P450 gene (*bcmD*) that was clustered with genes encoding five 2-oxoglutarate (2OG)-dependent dioxygenases (*bcmB*, *bcmC*, *bcmE*, *bcmF*, and *bcmG*), a gene encoding a major facilitator superfamily (MFS) transporter (*bcmT*), and a CPDS gene (*bcmA*) that is below the antiSMASH conserved domain detection limit for CDPSs (Fig. 1B and Table S2). Both P450s and 2OG-dependent dioxygenases are capable of catalyzing the regiospecific and stereospecific oxidation of nonactivated C—H bonds (22–24), while MFS transporters often function as drug efflux pumps and can confer antibiotic resistance (25, 26).

The putative CDPS (pfam16715) BcmA has multiple homologs (>45% identity) in other Actinobacteria and, notably, in various Pseudomonas aeruginosa strains. Interestingly, a homolog from P. aeruginosa (accession no. WP_003158562.1) was previously shown to catalyze the *in vitro* synthesis of cIL (27), and BcmA contains almost all the same specificity-determining binding pocket residues as WP_003158562.1 (Fig. S1). Surprisingly, the five 2OG-dependent dioxygenases encoded in the cluster share only moderate sequence identity (33 to 45%). In total, the gene cluster encodes six oxidative enzymes, which is consistent with the number of modifications required to convert cIL into BCM.

### Heterologous expression of the *bcm* gene cluster.

**FIG 2:**
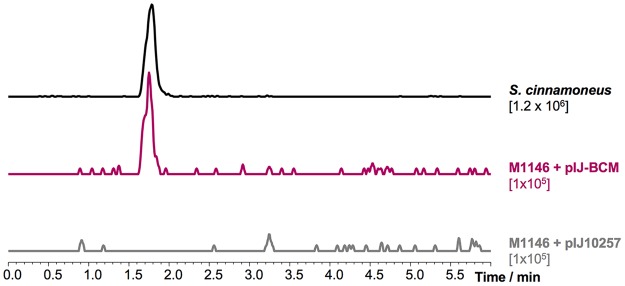
Heterologous expression of the *bcm* gene cluster from S. cinnamoneus in S. coelicolor M1146. Extracted ion chromatograms (EICs) of bicyclomycin (*m/z* 285.11, [M-H_2_O+H]^+^) in S. cinnamoneus, S. coelicolor M1146 expressing the *bcm* cluster, and S. coelicolor M1146 containing empty vector. The intensity scale of each EIC is noted under the corresponding label.

To test whether the identified gene cluster was indeed responsible and sufficient for the biosynthesis of BCM, a 7-kb region spanning *bcmA* to *bcmG* (*bcmA–G*) was PCR amplified and cloned into the ΦBT1 integrative vector pIJ10257 (28) by Gibson assembly (29) to generate pIJ-BCM. This places the constitutive promoter ermE*p before *bcmA*, which we anticipated would promote the expression of all *bcm* genes, as they are tightly clustered on the same strand. The putative transporter gene *bcmT* was not included on the basis that several homologs of this gene, as well as a homolog of the reported BCM resistance gene (30), are present in the Streptomyces coelicolor genome. pIJ-BCM was introduced into S. coelicolor M1146 and M1152 (31) via intergeneric conjugation. Liquid chromatography-tandem mass spectrometry (LC-MS^2^) analysis of cultures of the resulting strains yielded a peak of *m/z* 285.11 not present in the control strains (Fig. 2), which had an identical retention time and multiple-reaction monitoring (MRM) profile (*m/z* 211.05, *m/z* 193.2, *m/z* 108.4, and *m/z* 81.9; Fig. S2) to BCM produced by S. cinnamoneus, as well as a pure BCM standard, and corresponds to [BCM-H_2_O+H]^+^. This unambiguously confirmed that this was the BCM biosynthetic gene cluster. Our result agrees with those of recent studies by Patteson et al. (32) and Meng et al. (33) who, in parallel with our study, have reconstituted *in vitro* the functions of the CDPS and the oxidative steps in the S. cinnamoneus pathway.

### Identification and heterologous expression of a *bcm* gene cluster from Pseudomonas aeruginosa.

During our bioinformatic analysis of the S. cinnamoneus *bcm* gene cluster, it became clear that entire *bcm*-like gene clusters with an apparently identical organization of *bcmA–G* genes were present in a variety of Gram-negative and Gram-positive bacterial species and in particular in multiple P. aeruginosa strains. This distribution of such a conserved antibiotic gene cluster is very rare and prompted us to investigate whether these highly similar gene clusters actually make identical products. As a representative example, P. aeruginosa SCV20265 was therefore investigated for its ability to produce BCM. This strain is a well-studied (34–36) small-colony variant of the opportunistic pathogen isolated from the lung of a patient with cystic fibrosis (37) and is considered a reference strain in antibiotic resistance studies (38). The P. aeruginosa SCV20265 *bcm*-like gene cluster encodes proteins with sequence identities of between 30 and 56% compared to their Streptomyces counterparts. An MFS transporter is also encoded in this cluster but is at the end of the *bcmA–G* operon instead of preceding *bcmA* (Fig. 1B and Table S2).

**FIG 3:**
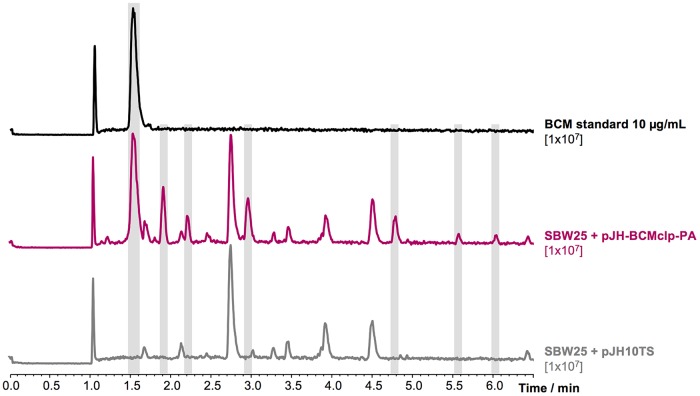
Heterologous expression of the *bcm* cluster from P. aeruginosa in P. fluorescens SBW25. Base peak chromatograms of a bicyclomycin standard, P. fluorescens SBW25 expressing the *bcm* cluster, and P. fluorescens SBW25 containing empty vector. The intensity scale of the chromatograms is noted under the corresponding labels. Compounds produced by the heterologous expression strain but not found in the control strain are highlighted in gray.

No BCM production was detected in cultures of P. aeruginosa SCV20265 in a range of production media, so heterologous expression of the gene cluster was carried out to determine whether the pathway is functional. The putative *bcm* cluster (including *bcmT*) was PCR amplified from SCV20265 genomic DNA (gDNA) and cloned into pJH10TS (39, 40), which places the putative *bcm* operon under the control of the synthetic promoter Ptac. Pseudomonas fluorescens SBW25 was transformed with the resulting plasmid (pJH-BCMclp-PA). Several clones of this heterologous expression strain were cultured in the same set of production media as P. aeruginosa and assessed for their ability to produce BCM. LC-MS^2^ analysis revealed that P. fluorescens SBW25/pJH-BCMclp-PA efficiently produces BCM after 14 h of growth (Fig. 3). The putative BCM detected in these samples exhibited the same retention time, mass, and fragmentation profile as a pure BCM standard, including MS signals of *m/z* 285.11, as observed previously, and *m/z* 325.10, corresponding to [BCM+Na]^+^ (Fig. 3, S3, and S4). This result is consistent with parallel work from Patteson et al. (32), but this does not preclude the possibility of variation in stereochemistry at one or more positions in the molecule. We therefore scaled up production, purified the compound, and subjected it to nuclear magnetic resonance (NMR) analysis (^1^H, ^13^C, correlation spectroscopy [COSY], heteronuclear multiple bond correlation [HMBC], and heteronuclear single-quantum correlation [HSQC]), which provided identical spectra (Fig. S5 to S10 and Table S4) to authentic BCM reported previously (41). Pseudomonas-produced BCM also had the same optical rotation as a BCM standard, confirming that they are stereochemically identical.

One of the most efficient media for BCM production in P. fluorescens was synthetic cystic fibrosis medium (SCFM), which mimics the salt and amino acid composition from cystic fibrosis sputum samples (42). The composition of this medium was simplified to generate bicyclomycin production medium (BCMM), in which cultures of P. fluorescens SBW25/pJH-BCMclp-PA provided BCM yields of 34.5 ± 2.1 mg/liter in only 14 h. Interestingly, we could detect at least six additional compounds in the heterologous expression strain in comparison to a negative-control strain harboring empty pJH10TS (Fig. 3, S3, and S4). All of these compounds have masses compatible with BCM-like compounds (Table S3), and some have BCM-like MS^2^ fragmentation patterns, such as a loss of 74.04 Da that corresponds to fragmentation of the oxidized leucine side chain (Fig. S4). This production profile makes P. fluorescens a promising BCM production system compared to the complex media and longer incubation times required to produce BCM in Streptomyces species, the current source of commercially available BCM. In contrast, we could not detect any BCM-like molecules in cultures of wild-type P. aeruginosa SCV20265, suggesting that additional factors are required to activate the expression of an otherwise-functional gene cluster.

### Organization, taxonomic distribution, and phylogeny of the *bcm* cluster.

The presence of seven contiguous biosynthetic genes that make the same antibiotic in both Gram-positive and Gram-negative bacteria was a fascinating result. The production of the same compound in such distantly related organisms (bacteria that are evolutionarily at least 1 billion years apart [43]) is incredibly rare but not unprecedented (44). To investigate this unusual result, a BLASTP search using BcmA was used to identify putative *bcm* gene clusters (*bcmA–G*) in sequenced bacterial genomes. In total, 724 candidates were identified, where 31 are found in a variety of taxa, and the remaining sequences all come from Pseudomonas species, in particular, P. aeruginosa. This initial data set was filtered (see Materials and Methods) to generate a final data set for phylogenetic analysis containing 374 *bcm*-like gene clusters (Data Set S1). Analysis of this data set showed that *bcm*-like gene clusters are also found in seven other sequenced Streptomyces species besides S. cinnamoneus, as well as 20 Mycobacterium chelonae strains, Williamsia herbipolensis (order Corynebacteriales), Actinokineospora spheciospongiae (order Pseudonocardiales), and the Gram-negative bacteria Burkholderia plantarii and Tistrella mobilis (from Betaproteobacteria and Alphaproteobacteria, respectively). Furthermore, a fragmented *bcm*-like gene cluster was identified in Photorhabdus temperata (Gammaproteobacteria) by BLAST analysis of BcmA and the P450 BcmD. This cluster is split across two different contigs (accession numbers NZ_AYSJ01000007 and NZ_AYSJ01000009), where it is accompanied by transposase genes and therefore was not included in our data set.

Most *bcm* gene clusters from Gram-positive bacteria share the same gene organization, with *bcmT* in an opposite orientation upstream of *bcmA*, whereas in all the Gram-negative bacteria (and Actinokineospora), *bcmT* is downstream of *bcmG* and in the same orientation as the rest of the cluster. Streptomyces ossamyceticus is the only representative that lacks a transporter gene immediately adjacent to the biosynthetic genes. Additionally, the MFS transporters in gene clusters from Gram-positive bacteria only share 27 to 30% sequence identity (approximately 40% coverage) with MFS transporters from Gram-negative gene clusters, suggesting that the transporters have been recruited independently from the rest of the cluster in these distant bacteria.

**FIG 4:**
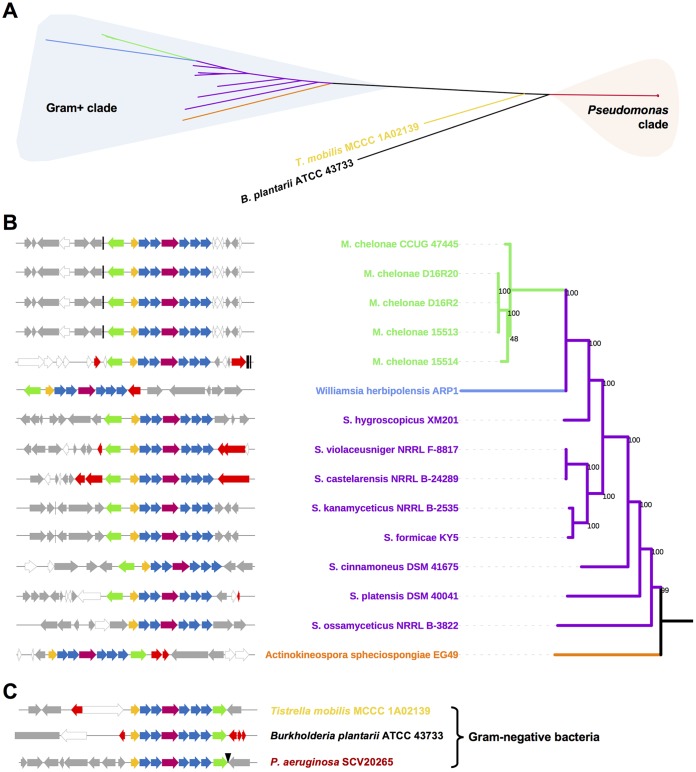
Phylogeny and genetic context of the *bcm* gene clusters. (A) Unrooted maximum likelihood tree of the nucleotide sequences from the *bcm* gene clusters identified in this work. Branches are color-coded by genus, and major clades are highlighted. (B) Detailed view of the phylogeny and genetic context of the *bcm* clusters in Gram-positive bacteria. Bootstrap support values for the phylogeny are shown at the base of each branch, and the genetic context of each cluster (color-coded as in Fig. 1B) is shown for each branch of the tree. Flanking genes are color-coded gray if they encode proteins with conserved domains, white for hypothetical proteins with no conserved domains, and red for proteins related to mobile genetic elements (see Table S5 for details). Vertical black lines represent tRNA genes. (C) Genetic context of the *bcm* clusters in Gram-negative bacteria. The black triangle represents an *att*Tn*7* site.

All the *bcm* gene clusters identified in this work were analyzed phylogenetically by constructing a maximum likelihood tree from the nucleotide sequence spanning *bcmA–G*. This showed that their evolutionary relationship correlates with bacterial genera (Fig. 4A). Clusters from Gram-negative (particularly Pseudomonas) and Gram-positive bacteria are grouped in completely independent and distant clades, while the clusters from Burkholderia and *Tistrella* appear at intermediate points between these two groups. Within the Gram-positive clade, the clusters have a higher degree of divergence but are similarly grouped according to the classification of their native species, with the Williamsia gene cluster clustering with the M. chelonae gene clusters (these two genera belong to the order Corynebacteriales) (Fig. 4B). All P. aeruginosa gene clusters are ∼99% identical to each other (Fig. 4A), whereas the two most distantly related streptomycete gene clusters share 69% identity and 83% coverage.

### Mobile genetic elements associated with *bcm*-like gene clusters.

The contrast between the genetic conservation of the *bcm* gene cluster and its distribution across distantly related bacteria strongly implies that the *bcm* gene cluster has been horizontally transferred between them. The increased sequence divergence of the *bcm* gene clusters in Streptomyces species suggests that the gene cluster may have originated from this taxonomic group, although it is difficult to prove this hypothesis, as the gene clusters in all strains appear to have adapted to their hosts, making HGT difficult to infer. Despite the below-average GC content of the clusters (59.6% in P. aeruginosa SCV20265 and 70.8% in S. cinnamoneus) versus the genome averages (66.3% and 72.4%, respectively), the clusters were not predicted to be part of genomic islands in these strains when analyzed with IslandViewer4 (45).

However, analysis of the genomic context of *bcm* gene clusters in P. aeruginosa strains strongly supports an insertion hypothesis, since the genes that flank the cluster are contiguous in a number of P. aeruginosa strains that lack the cluster (Fig. S11). Most notably, *bcmT* is adjacent to the glucosamine-fructose-6-phosphate aminotransferase gene *glmS*, and the intergenic region that precedes *glmS* contains the specific attachment site for transposon Tn*7* (*att*Tn*7*) (46). Consistent with this observation, some strains that lack the *bcm* gene cluster (e.g., P. aeruginosa BL08) have mobile genetic elements integrated next to *glmS* (Fig. S11). Intriguingly, many strains, including the reference strain P. aeruginosa PAO1, contain an MFS transporter gene (PA5548 in PAO1) adjacent to *glmS* that is 99% identical with *bcmT* from SCV20265. This either indicates that the *bcm* gene cluster recently integrated next to an existing P. aeruginosa transporter or that a subset of strains lost the biosynthetic genes but retained a potential BCM resistance gene.

The *bcm*-like gene clusters in other Gram-negative bacteria (Burkholderia and *Tistrella* spp.) and most Gram-positive bacteria are located next to genes coding for integrases, transposases, and other genetic mobility elements (Fig. 4B and C and Table S5). For example, the mycobacterial clusters are found close to tRNA genes, and their flanking genes are syntenic in some Mycobacterium abscessus strains, whereas in other M. abscessus strains, these genes are separated by a cluster of phage-related genes (Fig. 4B and S12). In the streptomycetes, the clusters are integrated in different genomic locations, where they are also often associated with mobile genetic elements (Fig. 4B and Table S5). This observation strongly supports HGT of the cluster between these taxa as well.

### Diversity and geographical distribution of the *bcm* cluster in P. aeruginosa.

**FIG 5:**
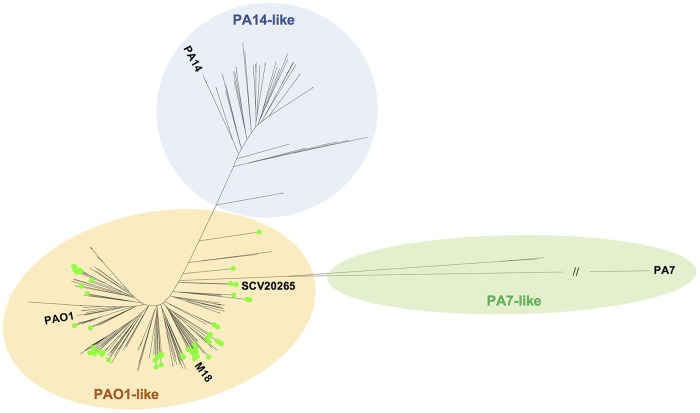
Distribution of the *bcm* gene cluster across P. aeruginosa isolates using a modified version of the unrooted maximum likelihood tree generated by Kos et al. (38). The PA14-like, PA7-like, and PAO1-like clades are color-coded, and a green dot signifies the presence of the *bcm* gene cluster. P. aeruginosa SCV20265 and multiple reference strains (PAO1, M18, PA7, and PA14) are also labeled.

The high sequence identity of the *bcm* gene cluster across hundreds of P. aeruginosa strains (Fig. 4A) along with its consistent genomic context (Fig. 4C) led us to question whether this cluster is truly widespread or only found in a small subset of P. aeruginosa strains that are overrepresented in sequence databases. P. aeruginosa isolates have been widely sequenced to evaluate pathogen diversity and evolution (38, 47, 48). As a result, large collections of sequenced clinical isolates are available in the databases, potentially constituting a biased data set that might lead to an overestimation of *bcm* gene cluster abundance and conservation. Most of the sequences in our final *bcm* data set come from well-characterized isolate collections. Among them, the Kos collection (38) provides a comprehensive survey of P. aeruginosa diversity, and the *bcm* gene cluster is present in nearly 20% of the isolates sequenced in this collection (74 out of the 390 isolates). To assess the phylogenetic diversity of these strains, we plotted the presence of the *bcm* gene cluster onto the Kos collection phylogenetic tree (38). Strikingly, this showed that nearly all of the *bcm*-positive strains are found in the PAO1 clade (Fig. 5), but these come from very diverse locations, including the United States, Mexico, Spain, France, Germany, China, Argentina, Brazil, Colombia, Croatia, and Israel, among others. This geographic diversity was further augmented by an analysis of all P. aeruginosa strains encoding the pathway (Data Set S1). We can therefore conclude that the *bcm* gene cluster is distributed globally but within a phylogenetically distinct subset of P. aeruginosa strains. Given this phylogenetic distribution, it is surprising to note that a *bcmT* gene is also found next to *glmS* in P. aeruginosa PA14 (Fig. S11), even though no isolates within the PA14 clade carry the *bcm* gene cluster.

### 2OG-dependent dioxygenase phylogeny.

An unusual feature of the *bcm* gene clusters is the presence of five 2OG-dependent dioxygenase genes. While it is possible that they originally arose by gene duplication events, the S. cinnamoneus 2OG-dependent dioxygenases only possess 33 to 45% sequence identity with each other (Fig. S13). We hypothesized that an analysis of the diversity of the *bcm* 2OG-dependent dioxygenases across multiple taxa could provide an insight into gene cluster evolution. We therefore constructed a maximum likelihood tree using protein sequences of every 2OG-dependent dioxygenase (BcmB, BcmC, BcmE, BcmF, and BcmG homologs) from both S. cinnamoneus and P. aeruginosa SCV20265, as well as from other selected P. aeruginosa strains and at least one representative from the other genera that contain *bcm*-like gene clusters.

**FIG 6:**
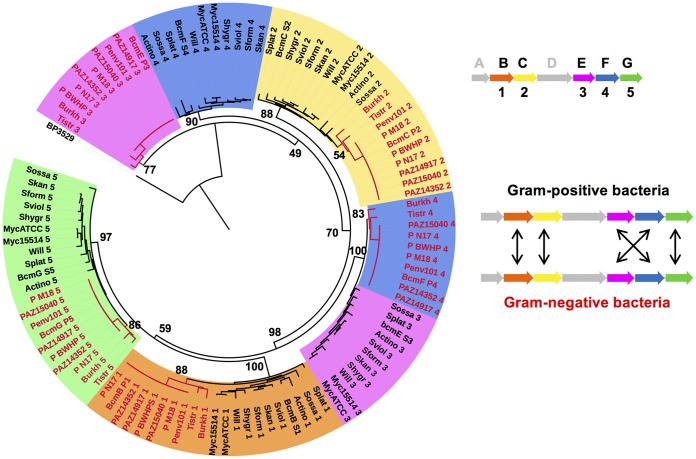
Maximum likelihood tree of the *bcm* 2-OG dioxygenases, including all representatives from Streptomyces, Actinokineospora (Actino), Williamsia (Will), Burkholderia (Burkh), and *Tistrella* (Tistr), two from Mycobacterium, and eight from Pseudomonas. Protein BP3529 from Bordetella pertussis was used as an outgroup (see Fig. S14 for an unrooted version of this tree). Background colors and numbering of the branch labels represent the position of a particular dioxygenase in the *bcm* gene cluster, as shown in the schematic representation. The taxonomic origins of each protein are indicated by their branch and label colors (black for Gram-positive and red for Gram-negative representatives). Bootstrap support values for the major branches are shown. The evolutionary relationships between *bcm* oxygenases from Gram-positive and Gram-negative bacteria are indicated by arrows in the diagram.

In contrast to the overall gene cluster phylogeny, the evolutionary relationship of the *bcm* oxidases correlates with their position in the cluster, as would be expected for a horizontally transferred unit (Fig. 6 and S14). BcmB, BcmC, and BcmG homologs group clearly in different clades, and within these clades the sequences from Gram-negative bacteria branch out from the Gram-positive subgroups, perhaps indicating the ancestral origin of these proteins. A surprising result was the unexpected phylogeny of the remaining two 2OG-dependent dioxygenases, BcmE and BcmF. These are clearly separated into two different clades: one containing BcmE from Gram-negative bacteria (BcmE−) and BcmF from Gram-positive bacteria (BcmF+), and one where BcmE+ groups with BcmF−. Within these two clades, Gram-positive and Gram-negative representatives are more distinct and bifurcate earlier than in the other clades (Fig. 6 and S14). The phylogenetic relationship between the 2OG-dependent dioxygenases strongly supports HGT of the cluster between taxa, although the BcmE/BcmF phylogeny indicates that the cluster may have undergone partial reorganization (Fig. 6). This intriguing result might mean that BcmE and BcmF fulfill inverse roles in Gram-positive and Gram-negative bacteria, and further experiments are necessary to test this hypothesis.

In summary, we demonstrate that the antibiotic BCM is a CDPS-derived natural product whose biosynthetic gene cluster is present in a diverse array of both Gram-positive and Gram-negative bacteria. This characterization was supported by heterologous expression of pathways from S. cinnamoneus and P. aeruginosa, where the pathway product was proven to be stereochemically identical to authentic BCM. We have also showed that the previously orphan P. aeruginosa pathway is a promising system for the production of BCM and related derivatives. The *bcm* cluster is dispersed across a number of taxonomically distant bacteria, including Alphaproteobacteria, Betaproteobacteria, and Gammaproteobacteria, as well as several families in Actinobacteria. The widespread presence of *bcmT* genes in P. aeruginosa (even those that lack the biosynthetic genes; Fig. S11), may explain why BCM is inactive toward P. aeruginosa (49), but further work is required to determine whether *bcmT* confers BCM resistance.

The presence of mobile genetic elements associated with the *bcm* gene cluster in many bacteria strongly supports the dissemination of this gene cluster via HGT, and the diversity of the gene cluster in Gram-positive bacteria suggests that it was subsequently transferred to Gram-negative bacteria, where two dioxygenase genes have apparently rearranged in the gene cluster and an alternative MFS transporter was acquired. However, the opposite direction of horizontal transfer cannot be ruled out. We are not aware of such a widespread distribution of any other specialized metabolite gene cluster, although there are examples of compounds that have been found in both Gram-positive and Gram-negative bacteria, such as pyochelin (50), the coronafacoyl phytotoxins (51), and furanomycin (52). Moreover, even in the cases where a similar compound is produced by distantly related bacteria, this can be achieved using different biosynthetic machinery. This is the case for the antibiotic fosfomycin, whose biosynthesis in Streptomyces and Pseudomonas spp. is catalyzed by distinct pathways that have undergone convergent evolution (53). Examples of highly conserved biosynthetic gene clusters between distantly related bacteria are rare, where one or more genes are different, such as the althiomycin gene clusters in Serratia marcescens and Myxococcus xanthus (54). A recent study by McDonald and Currie showed that it is very rare to find intact laterally transferred biosynthetic gene clusters, even between streptomycetes (55).

Given this distribution of *bcm* gene clusters, it will be interesting to determine the ecological role of BCM, especially given the abundance of functional pathways in pathogenic P. aeruginosa strains isolated from lungs, where adaptive evolutionary pressure would have led to the loss or decay of the cluster unless it conferred a competitive advantage (56). Antibacterial natural products can have roles in pathogen virulence, such as a bacteriocin produced by the pathogen Listeria monocytogenes that modifies intestinal microbiota to promote infection (57). In addition, given the horizontal transfer of the *bcm* gene cluster and its extensive association with mobile genetic elements, it is interesting to note that the transcription terminator Rho most strongly represses the transcription of horizontally acquired regions of genomes (58), an activity that would be specifically inhibited by BCM (7). It is known that phages recruit genes from bacteria that increase their fitness and that of their hosts (59, 60), and this may occur with the *bcm* gene cluster. These intriguing observations invite further work to be conducted to determine the natural role of BCM.

## MATERIALS AND METHODS

### Chemicals and molecular biology reagents.

Pharmamedia was obtained from Archer Daniels Midland Company. Antibiotics and all other medium components and reagents were purchased from Sigma-Aldrich. Bicyclomycin was purchased from BioAustralis Fine Chemicals (Australia). Enzymes were purchased from New England BioLabs unless otherwise specified, and molecular biology kits were purchased from Promega and GE Healthcare.

### Bacterial strains, plasmids, and culture conditions.

Escherichia coli, Streptomyces, and Pseudomonas strains, as well as plasmids and oligonucleotides used or generated in this work, are reported in Tables 1 and 2. S. cinnamoneus DSM 41675 was acquired from the German Collection of Microorganisms and Cell Cultures (DSMZ, Germany), P. aeruginosa SCV20265 was provided by Susanne Häussler (Helmholtz Centre for Infection Research, Germany), and pJH10TS was provided by Barrie Wilkinson (John Innes Centre, UK). E. coli and Pseudomonas strains were grown in lysogeny broth (LB) at 37°C (except for P. fluorescens SBW25, which is temperature sensitive and was grown at 28°C) and stored at −70°C in 50% glycerol stocks. Streptomyces strains were cultured in liquid tryptone soya broth (TSB; Oxoid) or solid soya flour mannitol (SFM) medium (61) at 28 to 30°C and stored at −70°C as 20% glycerol spore stocks.

**TABLE 1:** Strains and plasmids used or generated in this study

Strain or plasmid	Description^*a*^	Reference or source
Strains		
E. coli DH5α	F^−^ *endA1 glnV44 thi-1 recA1 relA1 gyrA96 deoR nupG purB20* ϕ80d*lacZ*ΔM15 Δ(*lacZYA-argF*)U169 hsdR17(r_K_^−^ m_K_^+^), λ^−^	80
E. coli ET12567	F^−^ *dam*-13::Tn*9 dcm-6 hsdM hsdR zjj-202*::Tn*10 recF143 galK2 galT22 ara-14 lacY1 xyl*-5 *leuB6 thi-1 tonA31 rpsL*136 *hisG4 tsx-78 mtl-1 glnV44*	81
S. cinnamoneus DSM 41675	Wild-type producer of bicyclomycin	1
S. coelicolor		
M1146	Δ*act* Δ*red* Δ*cpk* Δ*cda*	31
M1152	Δ*act* Δ*red* Δ*cpk* Δ*cda rpoB*[C1298T]	31
M1146/pIJ-BCM	M1146 containing pIJ-BCM	This study
M1152/pIJ-BCM	M1152 containing pIJ-BCM	This study
M1146/pIJ10257	M1146 containing pIJ10257	This study
M1152/pIJ10257	M1152 containing pIJ10257	This study
P. aeruginosa SCV20265	Clinical isolate from cystic fibrosis patient	35, 36
P. fluorescens		
SBW25	Environmental isolate	82
SBW25/pJH-BCMclp-PA	SBW25 containing pJH-BCMclp-PA	This study
SBW25/pJH10TS	SBW25 containing pJH10TS	This study
Plasmids		
pIJ10257	Streptomyces ΦBT1 integrative vector, Hyg^r^ ermE*p	28
pIJ-BCM	pIJ10257 containing *bcm* cluster from Streptomyces	This study
pUZ8002	Helper plasmid for intergeneric conjugation, Kan^r^	70
pJH10TS	Pseudomonas expression vector, Tc^r^, tac promoter	40
pJH-BCMclp-PA	pJH10TS containing *bcm* cluster from P. aeruginosa	This study

a Hyg^r^, hygromycin resistance; Kan^r^, kanamycin resistance; Tc^r^, tetracycline resistance.

**TABLE 2:** Primers used in this study

Primer name	Sequence (5′→3′)^*a*^	Use
pIJ-bcm_start	GGTAGGATCGTCTAGAACAGGAGGCCCCATATGTCGCTAGAAGCGCAGCTGATGGAGCCT	Amplification and assembly of Streptomyces bcm cluster in pIJ10257
pIJ-bcm_end	CCAAGCTTATGCAGGACTCTAGTTAATTAAAACCGGAACTGAGCGGATCCCCGTGGCTGA	
bcm-cdps_chk_fw	CTGATGGAGCCTCGGGAAGAACC	PCR verification of exconjugants
bcm-cdps_chk_rv	GCAGGCGCTCGTGGTAGTCG	PCR verification of exconjugants
BCM_seq_1	TCCACCTGAAAGGGCGATGAC	pIJ-BCM sequencing verification
BCM_seq_2	TCGTCATCAACTTCGGTCTGTCG	pIJ-BCM sequencing verification
BCM_seq_3	CTTCGTGACCGTCCTCTACATCG	pIJ-BCM sequencing verification
BCM_seq_4	GGTGGACAGCCTCGTGCCC	pIJ-BCM sequencing verification
BCM_seq_5	CCTGAGTCTGAAGAGGCACGC	pIJ-BCM sequencing verification
BCM_seq_6	GTCTCCACGGAACGGGCG	pIJ-BCM sequencing verification
BCM_seq_7	CGGCTACGAGATCCTCCACGA	pIJ-BCM sequencing verification
BCM_seq_8	CACAAGGACTCCGGCTGGG	pIJ-BCM sequencing verification
pJH-BCMclp_start	TAACTGCGCTAGCACCTCTCGAGGCATCATATGGCCAAAACCAGATCGACG	Amplification and assembly of Pseudomonas bcm cluster in pJH10TS
pJH-BCMcl_end	AGGCGGTCACGCTCTCCAGCGAGCTCTCTAGAAGCCGGGGCAGGCATGC	
pJH_chk_fw	TAATGTGTGGAATTGTGAGCGG	PCR verification of transformants
pJH_chk_rv	TGAGCCAAATGAGGCGGTC	PCR verification of transformants
BCM_PA_seq_1	GCGTAACTATTTCCTGGAGCACT	pJH-BCMclp-PA sequencing verification
BCM_PA_seq_2	TAACCTTCAATCACTATCGCCC	pJH-BCMclp-PA sequencing verification
BCM_PA_seq_3	GAACGGATGCACGAGATCGC	pJH-BCMclp-PA sequencing verification
BCM_PA_seq_4	TCTGGCTCGGAGACGACCTG	pJH-BCMclp-PA sequencing verification
BCM_PA_seq_5	GTAGTAGACAACCCGGAACAAGC	pJH-BCMclp-PA sequencing verification
BCM_PA_seq_6	CACAGGTGCCGACCAGGAC	pJH-BCMclp-PA sequencing verification
BCM_PA_seq_7	TCTTCGATCATCCAGACGGC	pJH-BCMclp-PA sequencing verification
BCM_PA_seq_8	CAACGACGACATCCTCCTCTG	pJH-BCMclp-PA sequencing verification
BCM_PA_seq_9	ATGCCCTGTTCGTGGATAGC	pJH-BCMclp-PA sequencing verification

a Restriction sites used for assembly are underlined.

The following media were used for bicyclomycin production experiments: Aizunensis production medium (AIZ), adapted from Miyamura et al. (62) (20 g/liter glucose, 20 g/liter soy flour, 2 g/liter Bacto peptone, 2 g/liter NaNO_3_, 1 g/liter KH_2_PO_4_, 0.5 g/liter MgSO_4_·7H_2_O, 0.5 g/liter KCl, 0.001 g/liter FeSO_4_·7H_2_O [pH 7.0]), and cinnamoneus production medium (CIN), adapted from Miyoshi et al. (1) (20 g/liter potato starch, 20 g/liter cotton seed meal [Pharmamedia], 10 g/liter soy flour, 5 g/liter MgSO_4_·7H_2_O, 10.9 g/liter KH_2_PO_4_, 2.85 Na_2_HPO_4_ [pH 6.8]; a solid version of CIN medium with 20 g/liter agar was used to grow S. cinnamoneus for reliable spore production). Synthetic cystic fibrosis medium (SCFM) was prepared following the recipe reported by Kamath and coworkers (42), and an alternative medium optimized for bicyclomycin production (BCMM) was developed from SCFM and comprised of the following (per liter): 6.5 ml of 0.2 M NaH_2_PO_4_, 6.25 ml of 0.2 M Na_2_HPO_4_, 0.348 ml of 1 M KNO_3_, 0.122 g NH_4_Cl, 1.114 g KCl, 3.03 g NaCl, 10 mM morpholinepropanesulfonic acid (MOPS), 16.09 ml of 100 mM l-leucine, 11.2 ml of 100 mM l-isoleucine, 6.33 ml of 100 mM l-methionine, 15.49 ml of 100 mM l-glutamic acid hydrochloride, 6.76 ml of 100 mM l-ornithine–HCl, 1.92 ml of 84 mM l-cystine (dissolved in 0.8 M HCl), and 2 ml of 3.6 mM FeSO_4_·7H_2_O, all in Milli-Q water. The solution was adjusted to pH 6.8, filter sterilized, and supplemented with 0.606 ml of 1 M MgCl_2_ and 1.754 ml of 1 M CaCl_2_ (sterilized separately). When necessary, antibiotics were added at the following concentrations: 50 μg/ml hygromycin, 50 μg/ml apramycin, 50 μg/ml kanamycin, 25 μg/ml chloramphenicol, 25 μg/ml nalidixic acid, and 12.5 μg/ml tetracycline.

### Genome sequencing, annotation, and bioinformatics analysis of S. cinnamoneus.

Genomic DNA of S. cinnamoneus DSM 41675 was isolated according to the salting-out protocol (61), subjected to a TruSeq PCR-free library preparation, and sequenced using Illumina MiSeq (600 cycles, 2 × 300 bp) at the DNA Sequencing Facility, Department of Biochemistry, University of Cambridge (UK). MinION Nanopore sequencing (Oxford Nanopore Technologies, UK) was carried out using the protocol below.

A single colony from S. cinnamoneus grown on solid CIN medium was used to inoculate 50 ml TSB, which was incubated at 28°C overnight with shaking at 250 rpm. 1 ml of this seed culture was used to inoculate a further 50 ml of TSB, which was again incubated at 28°C overnight with shaking at 250 rpm. DNA was extracted from 10 ml of this culture using the salting-out procedure described before (61) and resuspended in 5 ml Tris-EDTA (TE) buffer. DNA concentration was quantified using a Qubit 2.0 fluorometer (Life Technologies), and fragment length and DNA quality were assessed using the Agilent TapeStation 2200 (Agilent Technologies).

Genomic DNA (∼11 μg in 100 μl) was fragmented using a Covaris g-TUBE (Covaris, UK) centrifuged at 3,380 × *g* for 90 s 2 times to achieve a fragment distribution with a peak at ∼16 kb. The sequencing library was prepared using Oxford Nanopore Technologies Nanopore sequencing kit SQK-NSK007 (R9 version), according to the manufacturer's protocol (16 May 2016 version), starting at the end-prep step with ∼2.5 ng of DNA. Half (12 μl) of the library was loaded onto a FLO-Min104 (R9 version) flow cell and sequenced for ∼22 h using the script MinKNOW NC_48hr_Sequencing_Run_FLO-Min104.py. The flow cell was restarted after ∼7 h. The remaining 12 μl of the library was loaded after restarting the flow cell at 22 h. Sequencing was then run for a further 43 h. Base calling was performed using Metrichor Desktop Agent (version 1.107, 2D basecalling for SQK-NSK007).

The complete raw data set comprised 7,044,217 paired-end 301-bp Illumina MiSeq reads and 53,048 Nanopore MinION reads that passed quality control (QC). The Nanopore reads were extracted to fastq format using the poRe R package (63). For the Illumina-only assembly, SPAdes version 3.6.2 (64) was used with the *k*-mer flag set to -k 21,33,55,77,99,127. For the Nanopore-only assembly, Canu version 1.5 (65) was used with genome size of 7.0 m and the -nanopore-raw flag. For the hybrid Illumina/Nanopore assembly, SPAdes version 3.8.2 (66) was used, supplied with both data sets and with the -careful and -nanopore flags. Contigs with low sequence coverage were removed from the hybrid assembly. All assembly tasks were conducted using 16 central processing units (CPUs) on a 256-Gb compute node within the Norwich Bioscience Institutes (NBI) High Performance Computing (HPC) cluster. Genome assembly statistics are reported in Table S1. The hybrid assembly genome sequence was annotated using Prokka (67), which implements Prodigal (68) as an *orf* calling tool.

### Cloning the S. cinnamoneus bcm gene cluster.

The DNA region containing the *bcm* gene cluster was PCR amplified from S. cinnamoneus gDNA using primers pIJ-bcm_start and pIJ-bcm_end with Herculase II Fusion DNA polymerase (Agilent). The resulting 6,981-bp fragment was gel purified and inserted via Gibson assembly (29) into pIJ10257 (a ΦBT1 integrative and hygromycin-resistant vector [28]) linearized with NdeI and PacI to generate plasmid pIJ-BCM. To verify that the cluster sequence in this construct was correct, the plasmid was Sanger sequenced using primers BCM_seq_1 to BCM_seq_8. All other DNA isolation and manipulation techniques were performed according to standard procedures (69).

### Genetic manipulation of Streptomyces and heterologous expression of the *bcm* cluster.

Methylation-deficient E. coli ET12567 carrying the helper plasmid pUZ8002 (70) was transformed with pIJ-BCM by electroporation. This was employed as the donor strain in an intergeneric conjugation with S. coelicolor M1146 and M1152 (31), which was performed according to standard protocols (61). Exconjugants were screened by colony PCR with primers bcm-cdps_chk_fw and bcm-cdps_chk_rv to confirm plasmid integration. Control strains containing empty pIJ10257 were also generated using the same methodology.

### Cloning and expression of the P. aeruginosa bcm gene cluster.

Genomic DNA of P. aeruginosa SCV20265 was obtained using the FastDNA Spin kit for soil (MP Biomedicals). The DNA region containing genes *bcmA* to *bcmT* preceded by their own native promoter was PCR amplified using primers pJH-BCMclp_start and pJH-BCMcl_end with Herculase II Fusion DNA polymerase (Agilent). The resulting 8,604-bp fragment was gel purified and inserted via Gibson assembly (29) into pJH10TS (a derivative of the broad-host-range IncQ expression vector pJH10 carrying the synthetic Tac promoter [39, 40]) linearized with NdeI and XbaI to generate expression plasmid pJH-BCMclp-PA. This plasmid was verified by Sanger sequencing with primers BCM_PA_seq_1 to BCM_PA_seq_9 and introduced into P. fluorescens SBW25 via electroporation of freshly made competent cells, which were prepared as follows: two 1-ml aliquots of an overnight culture of P. fluorescens were centrifuged at 11,000 × *g* for 1 min, and the pellets were washed three times with 1 ml of HEPES buffer each, centrifuging at 11,000 × *g* for 1 min in every wash. The two pellets were then merged and resuspended in 100 μl HEPES buffer, and 2 μl of plasmid prep was added to the cell suspension, which was electroporated applying 2,500 V. After electroporation, the suspension was transferred to 1 ml of fresh LB and incubated with shaking at 28°C for 1 h, after which 100 μl of the mixture were plated onto an LB plate containing 12.5 μg/ml tetracycline. As a negative control, the empty vector pJH10TS (40) was also transformed into P. fluorescens SBW25. In order to verify the presence and sequence accuracy of the construct in P. fluorescens, colony PCR was carried out with transformants using primers pJH_chk_fw and pJH_chk_rv. For the positive clones selected for downstream work, pJH-BCMclp-PA was recovered and sequenced with primers BCM_PA_seq_1 to BCM_PA_seq_9.

### Production and LC-MS analysis of BCM.

Thirty microliters of a concentrated stock of S. cinnamoneus spores was used to inoculate 10 ml AIZ medium in 50-ml flasks, which were incubated at 28°C with shaking at 250 rpm for 3 days. Five hundred microliters of this seed culture was used to inoculate 7 ml of CIN medium in 50-ml Falcon tubes covered with foam bungs. These production cultures were incubated at 28°C with shaking at 250 rpm for 4 days. The same procedure was used for S. coelicolor M1146/pIJ-BCM and M1152/pIJ-BCM. For production in P. fluorescens, 20 μl of cell stocks was used to inoculate 10 ml SCFM in 30-ml universal polystyrene tubes. These cultures were grown overnight at 28°C with shaking at 250 rpm, with the screw caps slightly loose to allow aeration, and 400-μl aliquots were used to inoculate 10 ml BCMM in 50-ml Falcon tubes covered with foam bungs. Production cultures were incubated for 12 to 16 h at 28°C with shaking at 250 rpm.

For the analysis of BCM production, 1-ml production culture samples were centrifuged at 18,000 × *g* for 5 min. Five microliters of these samples was analyzed by LC-MS using a Luna Omega 1.6-μm Polar C18 column (50 mm by 2.1 mm, 100 Å; Phenomenex) connected to a Shimadzu Nexera X2 ultrahigh-performance liquid chromatography (UHPLC) eluting with a linear gradient of 0 to 35% methanol in water plus 0.1% formic acid over 6 min, with a flow rate of 0.5 ml/min. MS data were obtained using a Shimadzu ion-trap–time of flight (IT-TOF) mass spectrometer coupled to the UHPLC and analyzed using the LabSolutions software (Shimadzu). MS data were collected in positive mode over an *m/z* 200 to 2,000 range, with an ion accumulation window of 10 ms and automatic sensitivity control of 70% of the base peak. The curved desolvation line (CDL) temperature was 250°C, and the heat block temperature was 300°C. MS^2^ data were collected between *m/z* 90 and 2,000 in a data-dependent manner for parent ions between *m/z* 200 and 1,500, using a collision-induced dissociation energy of 50% and a precursor ion width of 3 Da. The instrument was calibrated using sodium trifluoroacetate cluster ions prior to every run.

Additional LC-MS analysis was carried out using a Waters Xevo TQ-S tandem LC-MS fitted with the aforementioned column and employing the same chromatographic method but injecting 1 μl of sample. A multiple-reaction monitoring (MRM) method for BCM identification and quantification was configured with the IntelliStart software (Waters) using pure BCM as a standard. MRM is based on the tracking of signature fragment ions (transitions) of a selected parent ion (determined with a true standard) to ensure the unambiguous and quantitative identification of a given molecule. For BCM, the following transitions were monitored over a dwell time of 0.01 s each (collision energies applied in each case are listed in parentheses): for the parent ion with *m/z* 285.11 [M-H_2_O+H]^+^, 211.04 (16 V), 193.28 (20 V), 108.13 (28 V), and 81.93 (34 V); and for the parent ion with *m/z* 325.10 [M+Na]^+^, 307.07 (16 V), 251.07 (16 V), 233.18 (20 V), and 215.96 (22 V). Data were acquired in positive electrospray mode with a capillary voltage of 3.9 kV, desolvation temperature of 500°C, gas flow of 900 liters/h, cone gas flow of 150 liters/h, and nebulizer set to 7.0 × 10^5^ Pa. LC-MS data were analyzed using the MassLynx software and the quantification tool QuanLynx (Waters). Xevo MS peak areas were used to determine BCM yields in comparison to a BCM standard.

For the accurate mass measurement of the BCM-like compounds, high-resolution mass spectra were acquired on a Synapt G2-Si mass spectrometer (Waters) operated in positive mode with a scan time of 0.5 s in the mass range of *m/z* 50 to 600. Five-microliter samples were injected onto a Luna Omega 1.6-μm Polar C18 column (50 mm by 2.1 mm, 100 Å; Phenomenex) and eluted with a linear gradient of 1 to 40% acetonitrile in water plus 0.1% formic acid over 7 min. Synapt G2-Si MS data were collected with the following parameters: capillary voltage, 2.5 kV; cone voltage, 40 V; source temperature, 120°C; and desolvation temperature, 350°C. Leu-enkephalin peptide was used to generate a dual lock-mass calibration with *m/z* of 278.1135 and *m/z* of 556.2766 measured every 30 s during the run.

### Isolation and characterization of BCM from Pseudomonas.

Four 2-liter flasks containing 500 ml of BCMM were each inoculated with 20 ml of SBW25/pJH-BCMclp-PA SCFM seed culture grown overnight. After 20 h of fermentation at 28°C with shaking at 250 rpm, the culture broth (approximately 2 liters) was separated from the cells by centrifugation to yield a cell-free supernatant (ca. 2 liters). The supernatant was lyophilized and then resuspended in distilled water (0.6 liters). This aqueous solution was extracted with ethyl acetate (3 × 0.6 liters) and then with 1-butanol (3 × 3 liters). The solvent was removed to dryness from each extract to afford an ethyl acetate extract (0.014 g), a butanol extract (0.914 g), and an aqueous extract (7.06 g). LC-MS analysis determined that the target compounds were mainly in the butanol and the aqueous extracts. The aqueous extract (0.202 g) and all of the butanol extract were subjected to solid-phase chromatography (SPE) on a C18 cartridge (DSC-18, 20 ml) using a gradient of H_2_O-MeOH (100:0 to 80:20). Fractions containing BCM were combined and further purified by semipreparative HPLC (Phenomenex, Luna PFP [2], 250 mm by 10 mm, 5 μm; 2 ml/min, UV detection at 218 nm) using a linear gradient of MeOH-H_2_O from 2 to 35% MeOH over 35 min, yielding bicyclomycin (3.3 mg; retention time = 30.2 min). One-dimensional (1D) and 2D NMR spectra were recorded at a ^1^H resonance frequency of 400 MHz and a ^13^C resonance frequency of 100 MHz using a Bruker Avance 400 MHz NMR spectrometer operated using the TopSpin 2.0 software. Spectra were calibrated to the residual solvent signals of CD_3_SOCD_3_ with resonances at δ_H_ 2.50 and δ_C_ 39.52. Optical rotations were measured on a PerkinElmer polarimeter (model 341) using the sodium D line (589 nm) at 20°C. Commercial standard BCM was [α]^20^_D_ +42.8° (c 0.454, MeOH), and Pseudomonas BCM was [α]^20^_D_ +43.5° (c 0.091, MeOH).

### Identification of *bcm* gene clusters in sequenced bacteria.

The sequences used for the phylogenetic analyses performed in this work were retrieved as follows. A BLASTP search against the NCBI nonredundant protein sequence database was carried out using the CDPS BcmA from S. cinnamoneus as the query, and the accession numbers of the resulting 73 hits were retrieved. These accession numbers were then used as input for Batch Entrez (https://www.ncbi.nlm.nih.gov/sites/batchentrez) to retrieve all the genomic records associated with them in the RefSeq nucleotide database (i.e., genomic sequences containing the protein identifications [IDs] recovered from BLASTP). This yielded a total of 754 nucleotide records which were then analyzed using MultiGeneBlast (71) to ascertain which ones had the complete *bcm* gene cluster. Thirty of the 754 sequences were discarded on the basis that they did not contain the *bcm* gene cluster or that the sequence was truncated. Analysis of the metadata associated with the remaining records led to the exclusion of 217 P. aeruginosa sequences (accession numbers NZ_LCSU01000019.1 to NZ_LFDI01000014.1, ordered by taxonomic ID) in order to avoid overestimation of the cluster conservation, since they were all isolated from a single patient (72). An additional 134 P. aeruginosa sequences (accession numbers NZ_FRFJ01000027.1 to NZ_FUEJ01000078.1, ordered by taxonomic ID) were also excluded from the analysis, due to a lack of associated metadata that prevented an assessment of the diversity of the sample set. Finally, a sequence from accession number NZ_LLUU01000091.1 was also discarded due to the presence of a stretch of undetermined nucleotides (substituted with Ns) in the *bcm* gene cluster. This resulted in a final data set of 374 sequences: 372 putative *bcm* gene clusters (Data Set S1) plus the gene clusters from S. cinnamoneus DSM 41675 and P. aeruginosa SCV20265. For the downstream formatting of the data set sequences, scripts or programs that could be run in parallel to process multiple inputs were run via GNU Parallel (73).

### Phylogenetic analysis of the *bcm* gene cluster.

Nucleotide sequences of the 374 data set clusters were trimmed to span a nucleotide region from 200 bp upstream of the start of *bcmA* to 200 bp downstream of the end of *bcmG* (average length, 7,224 bp). Phylogenetic analyses were carried out using MUSCLE and RAxML, which were used through the CIPRES science gateway (74) and T-REX (75), and the trees were visualized and edited using iTOL (76).

The 374 trimmed *bcm* gene cluster sequences were aligned using MUSCLE (77) with the following parameters: muscle -in infile.fasta -seqtype dna -maxiters 2 -maxmb 30000000 -log logfile.txt -verbose -weight1 clustalw -cluster1 upgmb -sueff 0.1 -root1 pseudo -maxtrees 1 -weight2 clustalw -cluster2 upgmb -sueff 0.1 -root2 pseudo -objscore sp -noanchors -phyiout outputi.phy.

The resulting PHYLIP interleaved output file was then used to generate a maximum likelihood phylogenetic tree using RAxML (78). The program was configured to perform rapid bootstrapping (BS) with up to a maximum 1,000 BS replicate searches (or until convergence was reached), followed by a maximum likelihood search to identify the best tree, with the following input parameters: Raxml -T 4 -N autoMRE -n correctorientcluster -s infile.txt -c 25 -m GTRCAT -p 12345 -k -f a -x 12345.

During the phylogenetic analysis with RAxML, 225 sequences were found to be absolutely identical and were subsequently removed to allow for a streamlined analysis of cluster phylogeny. After the analysis, a sequence with accession number NZ_LLQO01000184.1 was also found to be truncated and was eliminated from the phylogenetic tree, which contained 148 nonredundant entries.

For the phylogenetic analyses of the 2-OG-dependent dioxygenases, the amino acid sequences of BcmB, BcmC, BcmE, BcmF, and BcmG from S. cinnamoneus, P. aeruginosa SCV20265, and a strain subset including all representatives from Streptomyces, Actinokineospora, Williamsia, Burkholderia, and *Tistrella* spp., as well as two from Mycobacterium and seven from Pseudomonas, were retrieved and aligned with MUSCLE (with same parameters as before except for -seqtype protein -hydro 5 -hydrofactor 1.2), and a maximum likelihood phylogenetic tree was generated with RAxML using the model -m PROTGAMMABLOSUM62, including protein BP3529 from Bordetella pertussis (accession no. P0A3X2.1) as an outgroup.

### Analysis of the genomic context of the *bcm* gene cluster.

For all of the sequences containing the *bcm* gene cluster, a 20-kb region around BcmA was retrieved and reannotated using Prokka. A subset of these sequences (all Gram-positive bacteria, plus Burkholderia, *Tistrella*, and several Pseudomonas strains) were analyzed for conserved domains using CDD at NCBI (79), and mobile genetic elements were identified by manual analysis.

### Accession number(s).

The genome sequence of S. cinnamoneus DSM 41675 has been deposited at DDBJ/ENA/GenBank (https://www.ncbi.nlm.nih.gov/GenBank/) under the accession no. PKFQ00000000 (BioProject PRJNA423036). The version described in this paper is version PKFQ01000000. The raw read data have been deposited at EBI ENA with the accession no. PRJEB24738.

## Supplementary Material

Supplemental material

## References

[1] Miyoshi T, Miyairi N, Aoki H, Kohsaka M, Sakai H 1972 Bicyclomycin, a new antibiotic. I. Taxonomy, isolation and characterization J Antibiot (Tokyo) 25:569–575. 4648311 10.7164/antibiotics.25.569

[2] Miyamura S, Ogasawara N, Otsuka H, Niwayama S, Tanaka H 1973 Antibiotic 5879 produced by *Streptomyces aizunensis*, identical with bicyclomycin. J Antibiot (Tokyo) 26:479–484. doi:10.7164/antibiotics.26.479. 4792062

[3] Ochi K, Saito Y, Umehara K, Ueda I, Kohsaka M 1984 Restoration of aerial mycelium and antibiotic production in a *Streptomyces griseoflavus* arginine auxotroph. Microbiology 130:2007–2013. doi:10.1099/00221287-130-8-2007. 6470674

[4] Borthwick AD 2012 2,5-Diketopiperazines: synthesis, reactions, medicinal chemistry, and bioactive natural products. Chem Rev 112:3641–3716. doi:10.1021/cr200398y. 22575049

[5] Kamiya T, Maeno S, Hashimoto M, Mine Y 1972 Bicyclomycin, a new antibiotic. II. Structural elucidation and acyl derivatives. J Antibiot (Tokyo) 25:576–581. 4648312

[6] Skordalakes E, Brogan AP, Park BS, Kohn H, Berger JM 2005 Structural mechanism of inhibition of the Rho transcription termination factor by the antibiotic bicyclomycin. Structure 13:99–109. doi:10.1016/j.str.2004.10.013. 15642265

[7] Kohn H, Widger W 2005 The molecular basis for the mode of action of bicyclomycin. Curr Drug Targets Infect Disord 5:273–295. doi:10.2174/1568005054880136. 16181146

[8] Washburn RS, Gottesman ME 2011 Transcription termination maintains chromosome integrity. Proc Natl Acad Sci U S A 108:792–797. doi:10.1073/pnas.1009564108. 21183718 PMC3021005

[9] Harford PS, Murray BE, DuPont HL, Ericsson CD 1983 Bacteriological studies of the enteric flora of patients treated with bicozamycin (CGP 3543/E) for acute nonparasitic diarrhea. Antimicrob Agents Chemother 23:630–633. doi:10.1128/AAC.23.4.630. 6859842 PMC184716

[10] Malik M, Li L, Zhao X, Kerns RJ, Berger JM, Drlica K 2014 Lethal synergy involving bicyclomycin: an approach for reviving old antibiotics. J Antimicrob Chemother 69:3227–3235. doi:10.1093/jac/dku285. 25085655 PMC4228776

[11] Brogan AP, Widger WR, Bensadek D, Riba-Garcia I, Gaskell SJ, Kohn H 2005 Development of a technique to determine bicyclomycin-Rho binding and stoichiometry by isothermal titration calorimetry and mass spectrometry. J Am Chem Soc 127:2741–2751. doi:10.1021/ja046441q. 15725032

[12] Park B-S, Widger W, Kohn H 2006 Fluorine-substituted dihydrobicyclomycins: synthesis and biochemical and biological properties. Bioorg Med Chem 14:41–61. doi:10.1016/j.bmc.2005.07.075. 16185879

[13] Bradley EL, Herbert RB, Lawrie KWM, Khan JA, Moody CM, Young DW 1996 The biosynthesis of the *Streptomyces* antibiotic bicyclomycin. Tetrahedron Lett 37:6935–6938. doi:10.1016/0040-4039(96)01521-3.

[14] Gomez-Escribano JP, Alt S, Bibb MJ 2016 Next generation sequencing of actinobacteria for the discovery of novel natural products. Mar Drugs 14:78. doi:10.3390/md14040078. PMC4849082 27089350

[15] Jain M, Olsen HE, Paten B, Akeson M 2016 The Oxford Nanopore MinION: delivery of nanopore sequencing to the genomics community. Genome Biol 17:239. doi:10.1186/s13059-016-1103-0. 27887629 PMC5124260

[16] Weber T, Blin K, Duddela S, Krug D, Kim HU, Bruccoleri R, Lee SY, Fischbach MA, Müller R, Wohlleben W, Breitling R, Takano E, Medema MH 2015 antiSMASH 3.0–a comprehensive resource for the genome mining of biosynthetic gene clusters. Nucleic Acids Res 43:W237–W243. doi:10.1093/nar/gkv437. 25948579 PMC4489286

[17] Cramer RA, Gamcsik MP, Brooking RM, Najvar LK, Kirkpatrick WR, Patterson TF, Balibar CJ, Graybill JR, Perfect JR, Abraham SN, Steinbach WJ, Steinbach WJ 2006 Disruption of a nonribosomal peptide synthetase in *Aspergillus fumigatus* eliminates gliotoxin production. Eukaryot Cell 5:972–980. doi:10.1128/EC.00049-06. 16757745 PMC1489275

[18] King RR, Calhoun LA 2009 The thaxtomin phytotoxins: sources, synthesis, biosynthesis, biotransformation and biological activity. Phytochemistry 70:833–841. doi:10.1016/j.phytochem.2009.04.013. 19467551

[19] Gondry M, Sauguet L, Belin P, Thai R, Amouroux R, Tellier C, Tuphile K, Jacquet M, Braud S, Courçon M, Masson C, Dubois S, Lautru S, Lecoq A, Hashimoto S, Genet R, Pernodet J-L 2009 Cyclodipeptide synthases are a family of tRNA-dependent peptide bond–forming enzymes. Nat Chem Biol 5:414–420. doi:10.1038/nchembio.175. 19430487

[20] Bonnefond L, Arai T, Sakaguchi Y, Suzuki T, Ishitani R, Nureki O 2011 Structural basis for nonribosomal peptide synthesis by an aminoacyl-tRNA synthetase paralog. Proc Natl Acad Sci U S A 108:3912–3917. doi:10.1073/pnas.1019480108. 21325056 PMC3053990

[21] James ED, Knuckley B, Alqahtani N, Porwal S, Ban J, Karty JA, Viswanathan R, Lane AL 2016 Two distinct cyclodipeptide synthases from a marine actinomycete catalyze biosynthesis of the same diketopiperazine natural product. ACS Synth Biol 5:547–553. doi:10.1021/acssynbio.5b00120. 26641496

[22] Werck-Reichhart D, Feyereisen R 2000 Cytochromes P450: a success story. Genome Biol 1:reviews3003. 11178272 10.1186/gb-2000-1-6-reviews3003 PMC138896

[23] Farrow SC, Facchini PJ 2014 Functional diversity of 2-oxoglutarate/Fe(II)-dependent dioxygenases in plant metabolism. Front Plant Sci 5:524. doi:10.3389/fpls.2014.00524. 25346740 PMC4191161

[24] Martinez S, Hausinger RP 2015 Catalytic mechanisms of Fe(II)- and 2-oxoglutarate-dependent oxygenases. J Biol Chem 290:20702–20711. doi:10.1074/jbc.R115.648691. 26152721 PMC4543632

[25] Quistgaard EM, Low C, Guettou F, Nordlund P 2016 Understanding transport by the major facilitator superfamily (MFS): structures pave the way. Nat Rev Mol Cell Biol 17:123–132. doi:10.1038/nrm.2015.25. 26758938

[26] Kumar S, He G, Kakarla P, Shrestha U, Ranjana KC, Ranaweera I, Willmon TM, Barr SR, Hernandez AJ, Varela MF 2016 Bacterial multidrug efflux pumps of the major facilitator superfamily as targets for modulation. Infect Disord Drug Targets 16:28–43. doi:10.2174/1871526516666160407113848. 27052334

[27] Jacques IB, Moutiez M, Witwinowski J, Darbon E, Martel C, Seguin J, Favry E, Thai R, Lecoq A, Dubois S, Pernodet J-L, Gondry M, Belin P 2015 Analysis of 51 cyclodipeptide synthases reveals the basis for substrate specificity. Nat Chem Biol 11:721–727. doi:10.1038/nchembio.1868. 26236937

[28] Hong H-J, Hutchings MI, Hill LM, Buttner MJ 2005 The role of the novel Fem protein VanK in vancomycin resistance in *Streptomyces coelicolor*. J Biol Chem 280:13055–13061. doi:10.1074/jbc.M413801200. 15632111

[29] Gibson DG, Young L, Chuang R-Y, Venter JC, Hutchison CA, Smith HO 2009 Enzymatic assembly of DNA molecules up to several hundred kilobases. Nat Methods 6:343–345. doi:10.1038/nmeth.1318. 19363495

[30] Bentley J, Hyatt LS, Ainley K, Parish JH, Herbert RB, White GR 1993 Cloning and sequence analysis of an *Escherichia coli* gene conferring bicyclomycin resistance. Gene 127:117–120. doi:10.1016/0378-1119(93)90625-D. 8486276

[31] Gomez-Escribano JP, Bibb MJ 2011 Engineering *Streptomyces coelicolor* for heterologous expression of secondary metabolite gene clusters. Microb Biotechnol 4:207–215. doi:10.1111/j.1751-7915.2010.00219.x. 21342466 PMC3818861

[32] Patteson JB, Cai W, Johnson RA, Santa Maria KC, Li B 2017 Identification of the biosynthetic pathway for the antibiotic bicyclomycin. Biochemistry 57:61–65. doi:10.1021/acs.biochem.7b00943. 29053243 PMC5760335

[33] Meng S, Han W, Zhao J, Jian X-H, Pan H-X, Tang G-L 2017 A six-oxidase cascade for tandem C—H bond activation revealed by reconstitution of bicyclomycin biosynthesis. Angew Chem Int Ed 57:719–723. doi:10.1002/anie.201710529. 29194897

[34] Wehmhöner D, Häussler S, Tümmler B, Jänsch L, Bredenbruch F, Wehland J, Steinmetz I 2003 Inter- and intraclonal diversity of the *Pseudomonas aeruginosa* proteome manifests within the secretome. J Bacteriol 185:5807–5814. doi:10.1128/JB.185.19.5807-5814.2003. 13129952 PMC193958

[35] von Götz F, Häussler S, Jordan D, Saravanamuthu SS, Wehmhöner D, Strüssmann A, Lauber J, Attree I, Buer J, Tümmler B, Steinmetz I 2004 Expression analysis of a highly adherent and cytotoxic small colony variant of *Pseudomonas aeruginosa* isolated from a lung of a patient with cystic fibrosis. J Bacteriol 186:3837–3847. doi:10.1128/JB.186.12.3837-3847.2004. 15175297 PMC419954

[36] Eckweiler D, Bunk B, Spröer C, Overmann J, Häussler S 2014 Complete genome sequence of highly adherent *Pseudomonas aeruginosa* small-colony variant SCV20265. Genome Announc 2:e01232-13. doi:10.1128/genomeA.01232-13. 24459283 PMC3900915

[37] Häussler S, Ziegler I, Löttel A, Götz F v, Rohde M, Wehmhöhner D, Saravanamuthu S, Tümmler B, Steinmetz I 2003 Highly adherent small-colony variants of *Pseudomonas aeruginosa* in cystic fibrosis lung infection. J Med Microbiol 52:295–301. doi:10.1099/jmm.0.05069-0. 12676867

[38] Kos VN, Déraspe M, McLaughlin RE, Whiteaker JD, Roy PH, Alm RA, Corbeil J, Gardner H 2015 The resistome of *Pseudomonas aeruginosa* in relationship to phenotypic susceptibility. Antimicrob Agents Chemother 59:427–436. doi:10.1128/AAC.03954-14. 25367914 PMC4291382

[39] El-Sayed AK, Hothersall J, Cooper SM, Stephens E, Simpson TJ, Thomas CM 2003 Characterization of the mupirocin biosynthesis gene cluster from *Pseudomonas fluorescens* NCIMB 10586. Chem Biol 10:419–430. doi:10.1016/S1074-5521(03)00091-7. 12770824

[40] Scott TA, Heine D, Qin Z, Wilkinson B 2017 An l-threonine transaldolase is required for l-threo-β-hydroxy-α-amino acid assembly during obafluorin biosynthesis. Nat Commun 8:15935. doi:10.1038/ncomms15935. 28649989 PMC5490192

[41] Kohn H, Abuzar S, Korp JD, Zektzer AS, Martin GE 1988 Structural studies of bicyclomycin. J Heterocyclic Chem 25:1511–1517. doi:10.1002/jhet.5570250548.

[42] Kamath KS, Pascovici D, Penesyan A, Goel A, Venkatakrishnan V, Paulsen IT, Packer NH, Molloy MP 2016 *Pseudomonas aeruginosa* cell membrane protein expression from phenotypically diverse cystic fibrosis isolates demonstrates host-specific adaptations. J Proteome Res 15:2152–2163. doi:10.1021/acs.jproteome.6b00058. 27246823

[43] Marin J, Battistuzzi FU, Brown AC, Hedges SB 2017 The timetree of prokaryotes: new insights into their evolution and speciation. Mol Biol Evol 34:437–446. 27965376 10.1093/molbev/msw245

[44] Masschelein J, Jenner M, Challis GL 2017 Antibiotics from Gram-negative bacteria: a comprehensive overview and selected biosynthetic highlights. Nat Prod Rep 34:712–783. doi:10.1039/C7NP00010C. 28650032

[45] Bertelli C, Laird MR, Williams KP, Lau BY, Hoad G, Winsor GL, Brinkman FSL 2017 IslandViewer 4: expanded prediction of genomic islands for larger-scale datasets. Nucleic Acids Res 45:W30–W35. doi:10.1093/nar/gkx343. 28472413 PMC5570257

[46] Choi K-H, Gaynor JB, White KG, Lopez C, Bosio CM, Karkhoff-Schweizer RR, Schweizer HP 2005 A Tn*7*-based broad-range bacterial cloning and expression system. Nat Methods 2:443–448. doi:10.1038/nmeth765. 15908923

[47] van Belkum A, Soriaga LB, LaFave MC, Akella S, Veyrieras J-B, Barbu EM, Shortridge D, Blanc B, Hannum G, Zambardi G, Miller K, Enright MC, Mugnier N, Brami D, Schicklin S, Felderman M, Schwartz AS, Richardson TH, Peterson TC, Hubby B, Cady KC 2015 Phylogenetic distribution of CRISPR-Cas systems in antibiotic-resistant *Pseudomonas aeruginosa*. mBio 6:e01796-15. doi:10.1128/mBio.01796-15. 26604259 PMC4669384

[48] Freschi L, Jeukens J, Kukavica-Ibrulj I, Boyle B, Dupont M-J, Laroche J, Larose S, Maaroufi H, Fothergill JL, Moore M, Winsor GL, Aaron SD, Barbeau J, Bell SC, Burns JL, Camara M, Cantin A, Charette SJ, Dewar K, Déziel É Grimwood K, Hancock REW, Harrison JJ, Heeb S, Jelsbak L, Jia B, Kenna DT, Kidd TJ, Klockgether J, Lam JS, Lamont IL, Lewenza S, Loman N, Malouin F, Manos J, McArthur AG, McKeown J, Milot J, Naghra H, Nguyen D, Pereira SK, Perron GG, Pirnay J-P, Rainey PB, Rousseau S, Santos PM, Stephenson A, Taylor V, Turton JF, Waglechner N, 2015 Clinical utilization of genomics data produced by the international *Pseudomonas aeruginosa* consortium. Front Microbiol 6:1036. doi:10.3389/fmicb.2015.01036. 26483767 PMC4586430

[49] Nishida M, Mine Y, Matsubara T, Goto S, Kuwahara S 1972 Bicyclomycin, a new antibiotic. 3. *In vitro* and *in vivo* antimicrobial activity. J Antibiot (Tokyo) 25:582–593. 4567453

[50] Seipke RF, Song L, Bicz J, Laskaris P, Yaxley AM, Challis GL, Loria R 2011 The plant pathogen *Streptomyces scabies* 87-22 has a functional pyochelin biosynthetic pathway that is regulated by TetR- and AfsR-family proteins. Microbiology 157:2681–2693. doi:10.1099/mic.0.047977-0. 21757492

[51] Bown L, Li Y, Berrué F, Verhoeven JTP, Dufour SC, Bignell DRD 2017 Coronafacoyl phytotoxin biosynthesis and evolution in the common scab pathogen *Streptomyces scabiei*. Appl Environ Microbiol 83:e01169-17. doi:10.1128/AEM.01169-17. PMC5601335 28754703

[52] Trippe K, McPhail K, Armstrong D, Azevedo M, Banowetz G 2013 *Pseudomonas fluorescens* SBW25 produces furanomycin, a non-proteinogenic amino acid with selective antimicrobial properties. BMC Microbiol 13:111. doi:10.1186/1471-2180-13-111. 23688329 PMC3662646

[53] Kim SY, Ju K-S, Metcalf WW, Evans BS, Kuzuyama T, van der Donk WA 2012 Different biosynthetic pathways to fosfomycin in *Pseudomonas syringae* and *Streptomyces* species. Antimicrob Agents Chemother 56:4175–4183. doi:10.1128/AAC.06478-11. 22615277 PMC3421606

[54] Gerc AJ, Song L, Challis GL, Stanley-Wall NR, Coulthurst SJ 2012 The insect pathogen *Serratia marcescens* Db10 uses a hybrid non-ribosomal peptide synthetase-polyketide synthase to produce the antibiotic althiomycin. PLoS One 7:e44673. doi:10.1371/journal.pone.0044673. 23028578 PMC3445576

[55] McDonald BR, Currie CR 2017 Lateral gene transfer dynamics in the ancient bacterial genus *Streptomyces*. mBio 8:e00644-17. doi:10.1128/mBio.00644-17. 28588130 PMC5472806

[56] Winstanley C, O'Brien S, Brockhurst MA 2016 *Pseudomonas aeruginosa* evolutionary adaptation and diversification in cystic fibrosis chronic lung infections. Trends Microbiol 24:327–337. doi:10.1016/j.tim.2016.01.008. 26946977 PMC4854172

[57] Quereda JJ, Dussurget O, Nahori M-A, Ghozlane A, Volant S, Dillies M-A, Regnault B, Kennedy S, Mondot S, Villoing B, Cossart P, Pizarro-Cerda J 2016 Bacteriocin from epidemic *Listeria* strains alters the host intestinal microbiota to favor infection. Proc Natl Acad Sci U S A 113:5706–5711. doi:10.1073/pnas.1523899113. 27140611 PMC4878514

[58] Cardinale CJ, Washburn RS, Tadigotla VR, Brown LM, Gottesman ME, Nudler E 2008 Termination factor Rho and its cofactors NusA and NusG silence foreign DNA in *E. coli*. Science 320:935–938. doi:10.1126/science.1152763. 18487194 PMC4059013

[59] Lindell D, Sullivan MB, Johnson ZI, Tolonen AC, Rohwer F, Chisholm SW 2004 Transfer of photosynthesis genes to and from *Prochlorococcus* viruses. Proc Natl Acad Sci U S A 101:11013–11018. doi:10.1073/pnas.0401526101. 15256601 PMC503735

[60] Arnold ML 2006 Evolution through genetic exchange. Oxford University Press, Oxford, United Kingdom.

[61] Kieser T, Bibb MJ, Buttner MJ, Chater KF, Hopwood DA 2000 Practical Streptomyces genetics. John Innes Foundation. John Innes Foundation, Norwich, United Kingdom.

[62] Miyamura S, Ogasawara N, Otsuka H, Niwayama S, Tanaka H 1972 Antibiotic no. 5879, a new water-soluble antibiotic against Gram-negative bacteria. J Antibiot 25:610–612. doi:10.7164/antibiotics.25.610. 4648315

[63] Watson M, Thomson M, Risse J, Talbot R, Santoyo-Lopez J, Gharbi K, Blaxter M 2015 poRe: an R package for the visualization and analysis of nanopore sequencing data. Bioinformatics 31:114–115. doi:10.1093/bioinformatics/btu590. 25173419 PMC4271141

[64] Bankevich A, Nurk S, Antipov D, Gurevich AA, Dvorkin M, Kulikov AS, Lesin VM, Nikolenko SI, Pham S, Prjibelski AD, Pyshkin AV, Sirotkin AV, Vyahhi N, Tesler G, Alekseyev MA, Pevzner PA 2012 SPAdes: a new genome assembly algorithm and its applications to single-cell computing. J Comput Biol 19:455–477. doi:10.1089/cmb.2012.0021. 22506599 PMC3342519

[65] Koren S, Walenz BP, Berlin K, Miller JR, Bergman NH, Phillippy AM 2017 Canu: scalable and accurate long-read assembly via adaptive k-mer weighting and repeat separation. Genome Res 27:722–736. doi:10.1101/gr.215087.116. 28298431 PMC5411767

[66] Antipov D, Korobeynikov A, McLean JS, Pevzner PA 2016 HybridSPAdes: an algorithm for hybrid assembly of short and long reads. Bioinformatics 32:1009–1015. doi:10.1093/bioinformatics/btv688. 26589280 PMC4907386

[67] Seemann T 2014 Prokka: rapid prokaryotic genome annotation. Bioinformatics 30:2068–2069. doi:10.1093/bioinformatics/btu153. 24642063

[68] Hyatt D, Chen G-L, Locascio PF, Land ML, Larimer FW, Hauser LJ 2010 Prodigal: prokaryotic gene recognition and translation initiation site identification. BMC Bioinformatics 11:119. doi:10.1186/1471-2105-11-119. 20211023 PMC2848648

[69] Sambrook J, Fritsch EF, Maniatis T 1989 Molecular cloning: a laboratory manual, 2nd ed Cold Spring Harbor Laboratory Press, Cold Spring Harbor, NY.

[70] Paget MS, Chamberlin L, Atrih A, Foster SJ, Buttner MJ 1999 Evidence that the extracytoplasmic function sigma factor sigmaE is required for normal cell wall structure in *Streptomyces coelicolor* A3(2). J Bacteriol 181:204–211. 9864331 10.1128/jb.181.1.204-211.1999 PMC103550

[71] Medema MH, Takano E, Breitling R 2013 Detecting sequence homology at the gene cluster level with MultiGeneBlast. Mol Biol Evol 30:1218–1223. doi:10.1093/molbev/mst025. 23412913 PMC3670737

[72] Diaz Caballero J, Clark ST, Coburn B, Zhang Y, Wang PW, Donaldson SL, Tullis DE, Yau YCW, Waters VJ, Hwang DM, Guttman DS 2015 Selective sweeps and parallel pathoadaptation drive Pseudomonas aeruginosa evolution in the cystic fibrosis lung. mBio 6:e00981-15. doi:10.1128/mBio.00981-15. 26330513 PMC4556809

[73] Tange O 2011 GNU parallel: the command-line power tool. USENIX Mag 36:42–47.

[74] Miller MA, Pfeiffer W, Schwartz T 2011 The CIPRES science gateway: a community resource for phylogenetic analyses, article 41 *In* Proceedings of the 2011 TeraGrid conference: extreme digital discovery 18 to 21 July 2011, Salt Lake City, UT.

[75] Boc A, Diallo AB, Makarenkov V 2012 T-REX: a web server for inferring, validating and visualizing phylogenetic trees and networks. Nucleic Acids Res 40:W573–W579. doi:10.1093/nar/gks485. 22675075 PMC3394261

[76] Letunic I, Bork P 2016 Interactive tree of life (iTOL) v3: an online tool for the display and annotation of phylogenetic and other trees. Nucleic Acids Res 44:W242–W245. doi:10.1093/nar/gkw290. 27095192 PMC4987883

[77] Edgar RC 2004 MUSCLE: multiple sequence alignment with high accuracy and high throughput. Nucleic Acids Res 32:1792–1797. doi:10.1093/nar/gkh340. 15034147 PMC390337

[78] Stamatakis A 2014 RAxML version 8: a tool for phylogenetic analysis and post-analysis of large phylogenies. Bioinformatics 30:1312–1313. doi:10.1093/bioinformatics/btu033. 24451623 PMC3998144

[79] Marchler-Bauer A, Derbyshire MK, Gonzales NR, Lu S, Chitsaz F, Geer LY, Geer RC, He J, Gwadz M, Hurwitz DI, Lanczycki CJ, Lu F, Marchler GH, Song JS, Thanki N, Wang Z, Yamashita RA, Zhang D, Zheng C, Bryant SH 2015 CDD: NCBI's conserved domain database. Nucleic Acids Res 43:D222–D226. doi:10.1093/nar/gku1221. 25414356 PMC4383992

[80] Grant SG, Jessee J, Bloom FR, Hanahan D 1990 Differential plasmid rescue from transgenic mouse DNAs into *Escherichia coli* methylation-restriction mutants. Proc Natl Acad Sci U S A 87:4645–4649. 2162051 10.1073/pnas.87.12.4645 PMC54173

[81] MacNeil DJ, Gewain KM, Ruby CL, Dezeny G, Gibbons PH, MacNeil T 1992 Analysis of *Streptomyces avermitilis* genes required for avermectin biosynthesis utilizing a novel integration vector. Gene 111:61–68. doi:10.1016/0378-1119(92)90603-M. 1547955

[82] Rainey PB, Bailey MJ 1996 Physical and genetic map of the *Pseudomonas fluorescens* SBW25 chromosome. Mol Microbiol 19:521–533. doi:10.1046/j.1365-2958.1996.391926.x. 8830243

