# Supplementary material for: Discovery and Biosynthesis of the Antibiotic Bicyclomycin in Distantly Related Bacterial Classes
Source: Appl Environ Microbiol. 2018 Apr 16;84(9):e02828-17. doi: 10.1128/AEM.02828-17 (PMC5930311; doi:10.1128/AEM.02828-17)
Supplement: Supplemental material [file supp_84_9_e02828-17__index.html]

Supplemental material 

# Discovery and Biosynthesis of the Antibiotic Bicyclomycin in Distantly Related Bacterial Classes

## Supplemental material

- Supplemental file 1 -

  Statistics of the *S. cinnamoneus* DSM 41675 genome sequence assemblies (Table S1); *bcm* gene clusters (Table S2); alignment of a *P. aeruginosa* CDPS with BcmA from *S. cinnamoneus* (Fig. S1); MRM analysis of bicyclomycin from *S. cinnamoneus* (Fig. S2); MS and MS2 (Fig. S3) and LC-MS (Fig. S4) spectra; exact masses of the BCM-like compounds produced by *Pseudomonas* (Table S3); main 1H-13C HMBC correlations identified for bicyclomycin from *Pseudomonas* (Fig. S5); NMR data (Table S4 and Fig. S6 to S10); mobile genetic elements and tRNA genes surrounding the *bcm* cluster in different bacteria (Table S5); genomic context of the *bcm* cluster in *P. aeruginosa* (Fig. S11) and *Mycobacterium* (Fig. S12); alignment and percentage identity matrix of the *bcm* 2-OG/Fe dioxygenases from *S. cinnamoneus* (Fig. S13); unrooted version of the phylogenetic tree shown in Fig. 6 (Fig. S14).

  PDF, 2.4M
- Supplemental file 2 -

  Curated collection of sequenced strains with *bcm*-like gene clusters (Data Set S1).

  XLSX, 124K
