# Supplementary material for: Discovery and Biosynthesis of the Antibiotic Bicyclomycin in Distantly Related Bacterial Classes
Source: Appl Environ Microbiol. 2018 Apr 16;84(9):e02828-17. doi: 10.1128/AEM.02828-17 (PMC5930311; doi:10.1128/AEM.02828-17)
Supplement: Supplemental material [file AEM.02828-17_zam009188489s1.pdf]

SUPPLEMENTAL MATERIAL

Discovery and biosynthesis of the antibiotic bicyclomycin in distant bacterial classes

Natalia M. Vior, Rodney Lacret, Govind Chandra, Siobhán Dorai-Raj, Martin Trick, Andrew W. Truman

TABLE OF CONTENTS

Table S1. Statistics of the *S. cinnamoneus* DSM 41675 genome sequence assemblies.

Table S2. *bcm* gene clusters in *S. cinnamoneus* DSM 41675 and *P. aeruginosa* SCV20265.

Figure S1. Alignment of a *P. aeruginosa* CDPS with BcmA from *S. cinnamoneus.*

Figure S2. MRM analysis of bicyclomycin from *S. cinnamoneus*.

Figure S3. MS and MS^2 spectra for bicyclomycin from *Pseudomonas*.

Figure S4. Comparison of the LC-MS spectra of *P. fluorescens* SBW25-pJH-BCMclp-PA and an empty vector control.

Table S3. Exact masses of the BCM-like compounds produced by *Pseudomonas*.

Figure S5. Main ^{1}H-^{13}C HMBC correlations identified for bicyclomycin from *Pseudomonas*.

Table S4. ^{1}H and ^{13}C NMR data for BCM produced by *Pseudomonas* in CD_3SOCD_3.

Figure S6. 400 MHz ^{1}H NMR spectrum of BCM produced by *Pseudomonas* in CD_3SOCD_3.

Figure S7. 100 MHz ^{13}C NMR spectrum of BCM produced by *Pseudomonas* in CD_3SOCD_3.

Figure S8. 400 MHz ^{1}H-^{13}C HSQC NMR spectrum of BCM produced by *Pseudomonas* in CD_3SOCD_3.

Figure S9. 400 MHz ^{1}H-^{1}H COSY NMR spectrum of BCM produced by *Pseudomonas* in CD_3SOCD_3.

Figure S10. 400 MHz ^{1}H-^{13}C HMBC NMR spectrum of BCM produced by *Pseudomonas* in CD_3SOCD_3.

Table S5. Mobile genetic elements and tRNA genes surrounding the *bcm* cluster in different bacteria.

Figure S11. Genomic context of the *bcm* cluster in *P. aeruginosa.*

Figure S12. Genomic context of the *bcm* cluster in *Mycobacterium.*

Figure S13. Alignment and percentage identity matrix of the *bcm* 2-OG/Fe dioxygenases from *S. cinnamoneus*.

Figure S14. Unrooted version of the phylogenetic tree shown in Fig. 6.

	**Illumina only assembly**	**Nanopore only assembly**	**Hybrid assembly**
Sequence size	6,802,277	6,597,724	6,662,690
Number of contigs	415	4	2
GC content (%)	72.4	72.3	72.4
Longest contig size	896,920	4,033,611	6,463,510
N50 value	403,337	4,033,611	6,463,510
L50 value	6	1	1

Table S1. Statistics of the different *S. cinnamoneus* DSM 41675 genome sequence assemblies described in this work.

Gene	Locus tag in *S. cinnamoneus* DSM 41675	Locus tag in *P. aeruginosa* SCV20265	Gene product	Size (aa) in Scin/Paer
bcmA	CYQ11_26550	SCV20265_RS31155	tRNA-dependent cyclodipeptide synthase	241/244
bcmB	CYQ11_26545	SCV20265_RS31160	2OG-Fe(II) oxygenase	325/322
bcmC	CYQ11_26540	SCV20265_RS31165	2OG-Fe(II) oxygenase	305/313
bcmD	CYQ11_26535	SCV20265_RS31170	Cytochrome P450	488/476
bcmE	CYQ11_26530	SCV20265_RS31175	2OG-Fe(II) oxygenase	314/293
bcmF	CYQ11_26525	SCV20265_RS31180	2OG-Fe(II) oxygenase	296/315
bcmG	CYQ11_26520	SCV20265_RS31185	2OG-Fe(II) oxygenase	300/296
bcmT	CYQ11_26555	SCV20265_RS31190	MFS transporter	473/401

Table S2. *bcm* gene clusters in *S. cinnamoneus* DSM 41675 and *P. aeruginosa* SCV20265.

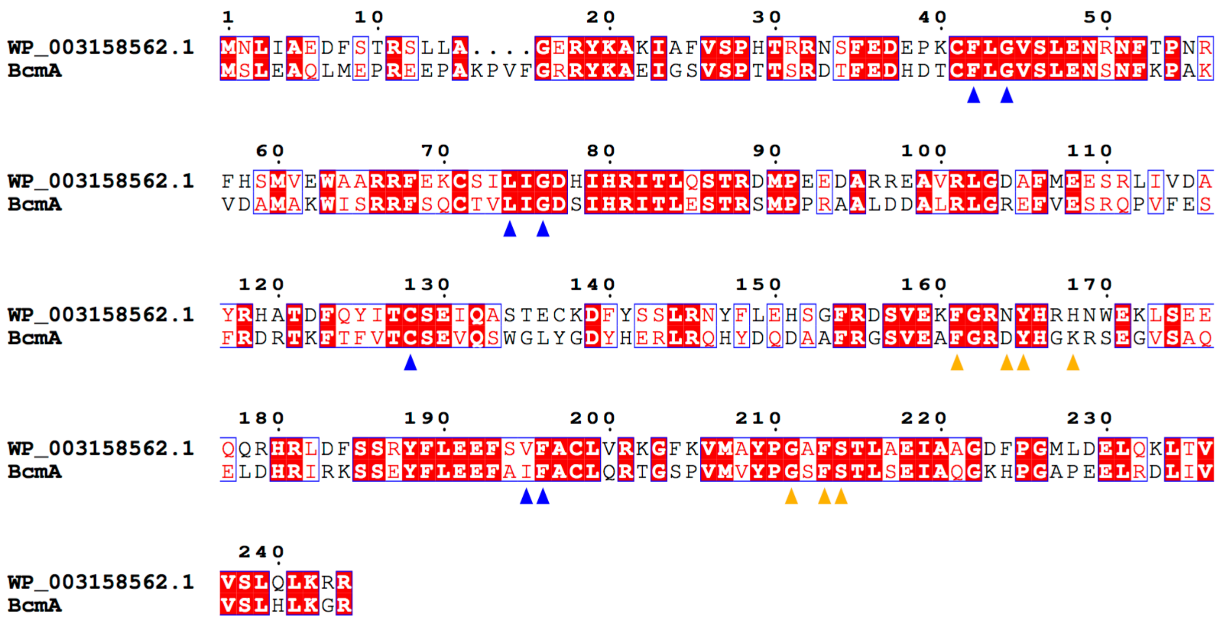

Figure S1. Alignment of a *P. aeruginosa* CDPS (WP_003158562.1) with selectivity for cyclo(L-Ile-L-Leu) and BcmA from *S. cinnamoneus*. Specificity-determining residues are highlight for P1 (blue triangles) and P2 (orange triangles) binding pockets, as determined by Jacques *et al.* (1). Identical residues have a red background while similar residues have red lettering. Figure generated using Espript 3.0 (2).

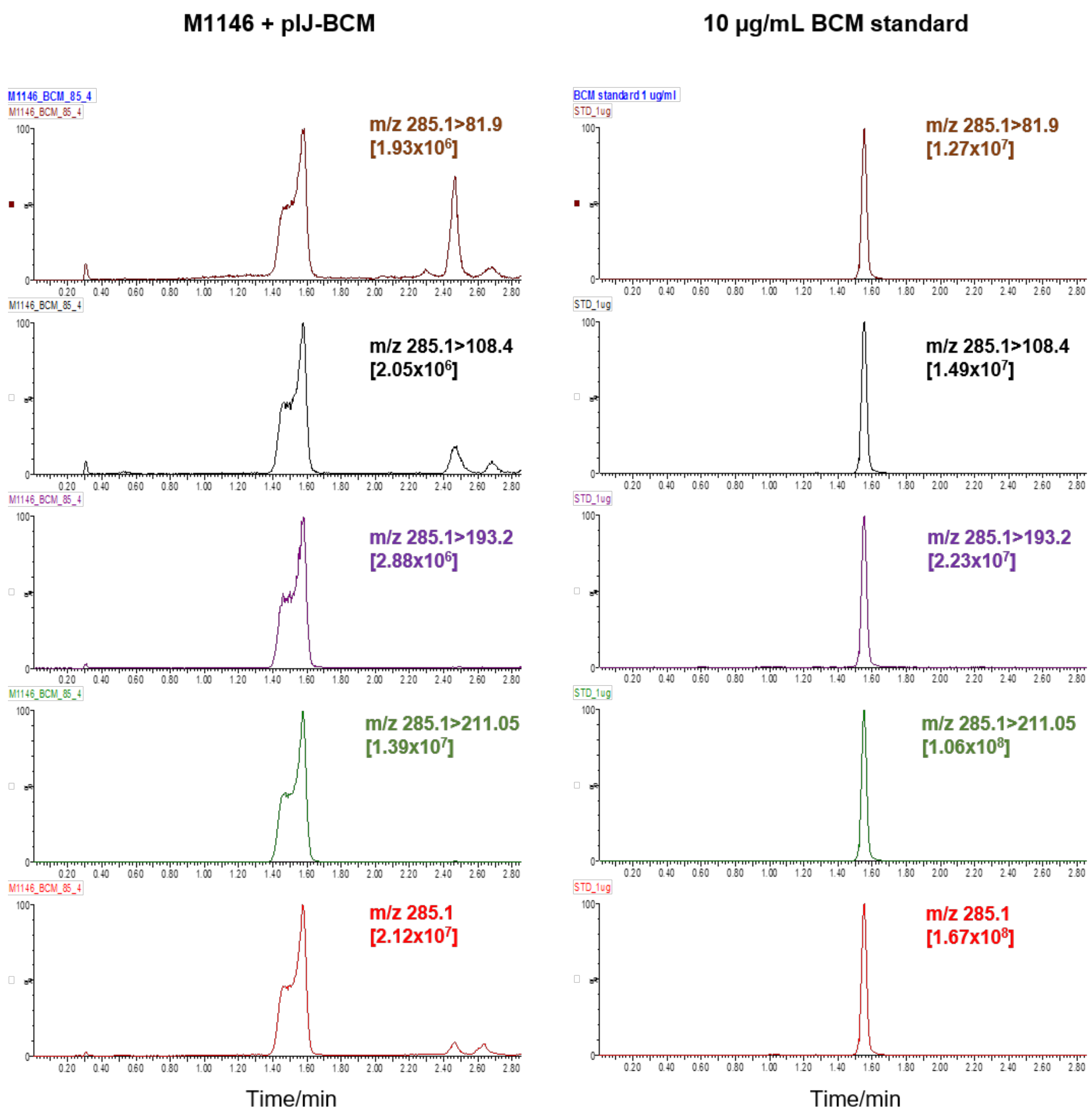

Figure S2. Multiple Reaction Monitoring (MRM) analysis of bicyclomycin (parent ion *m/z* 285.1, $[M-H_2O+H]^+$) produced by the heterologous expression strain M1146 carrying pIJ-BCM (left) compared with a pure BCM standard (right). The measured intensity for each monitored transition is indicated in brackets.

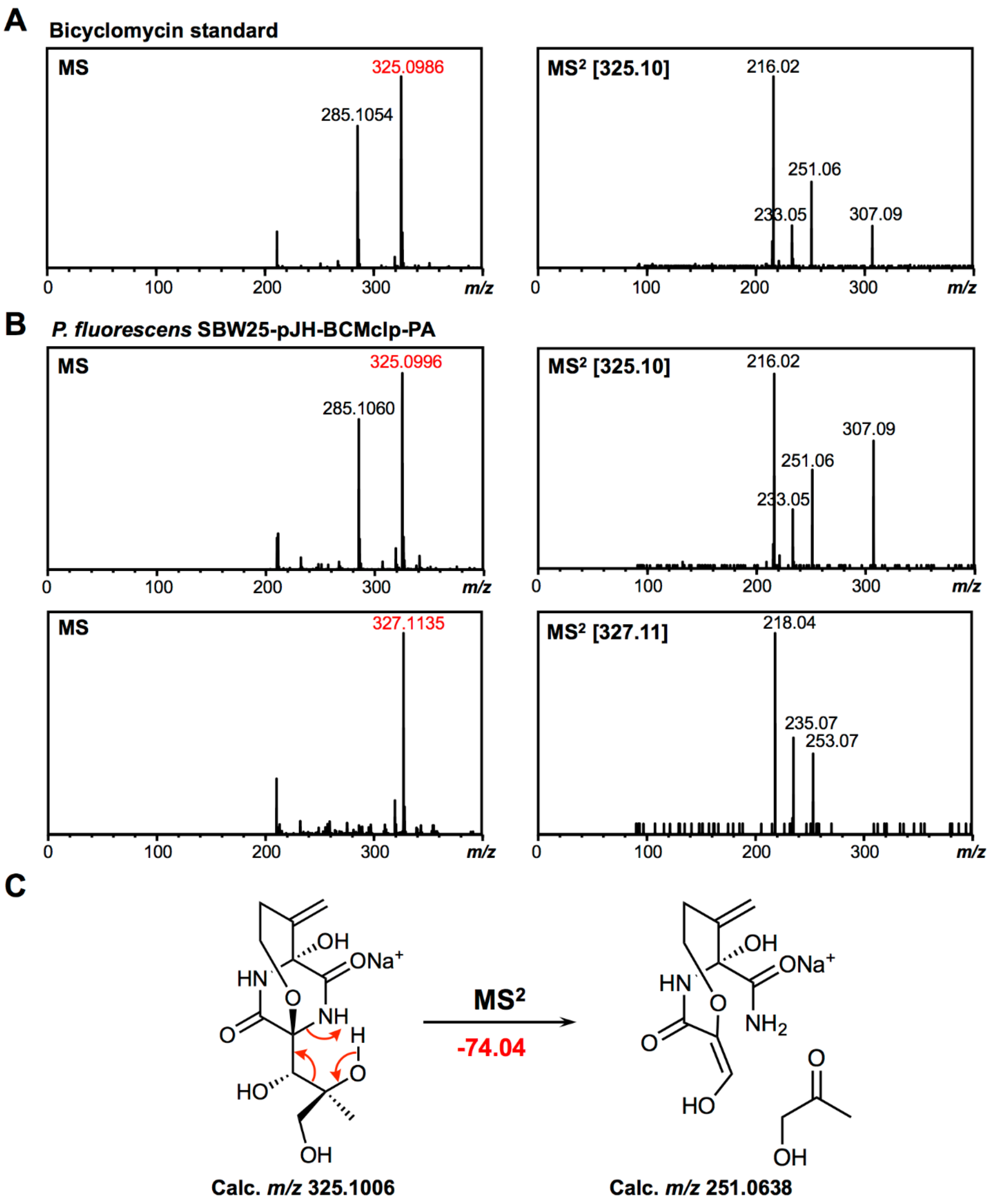

Figure S3. MS and MS^2 spectra for bicyclomycin. (A) MS spectrum for a commercial standard of BCM and MS^2 spectra for $[BCM+Na]^+$. (B) MS and MS^2 spectra for compounds with *m/z* 325.10 and *m/z* 327.11 produced by *P. fluorescens* SBW25-pJH-BCMclp-PA. (C) Proposed fragmentation mechanism leading to the loss of 74.04 Da. An alternative mechanism would involve opening of the ether instead of breaking the C-N bond.

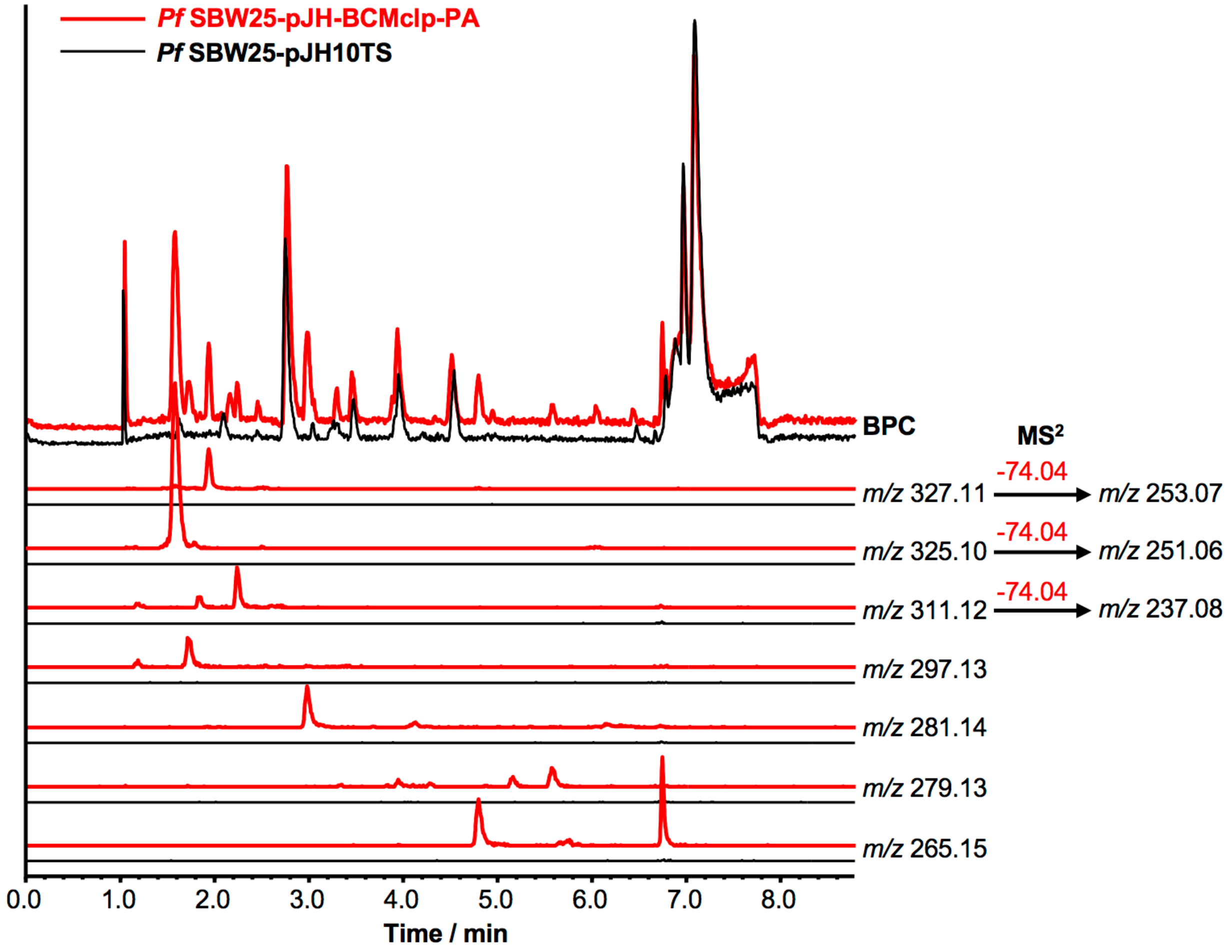

Figure S4. Comparison of the LC-MS spectra of *P. fluorescens* SBW25-pJH-BCMclp-PA (red) and an empty vector control (black). Extracted ion chromatograms are shown for putative $[M+Na]^+$ signals for the major metabolites that differed between these two fermentations (BPC = base peak chromatogram). Compounds with a BCM-like loss of 74.04 Da are highlighted.

**Predicted compound**	**[M+Na]$^+$ chemical formula**	**Pred. *m/z***	**Obs. *m/z***	**Error (ppm)**
**BCM**	$C_{12}H_{18}N_2NaO_7^+$	325.1006	325.1008	-0.62
**BCM+2H**	$C_{12}H_{20}N_2NaO_7^+$	327.1163	327.1163	0.00
**BCM+2H-O**	$C_{12}H_{22}N_2NaO_5^+$	311.1214	311.1208	1.93
**BCM+4H-2O**	$C_{12}H_{22}N_2NaO_5^+$	297.1421	297.1420	0.34
**BCM+4H-3O**	$C_{12}H_{22}N_2NaO_4^+$	281.1472	281.1467	1.78
**BCM+2H-3O**	$C_{12}H_{20}N_2NaO_4^+$	279.1315	279.1310	1.79
**BCM+4H-4O**	$C_{12}H_{22}N_2NaO_3^+$	265.1523	265.1520	1.13

Table S3. Exact masses of the BCM-like compounds produced by *P. fluorescens* SBW25-pJH-BCMclp-PA.

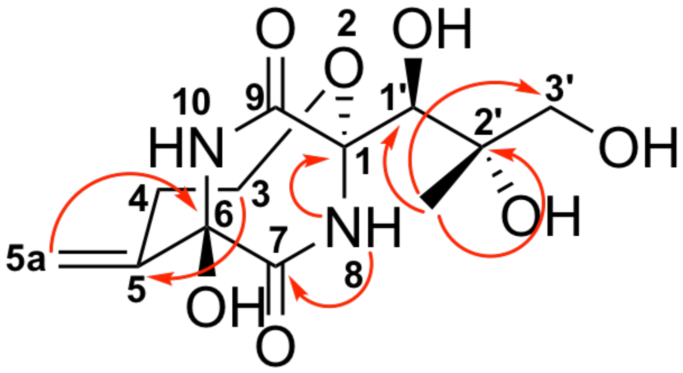

Figure S5. Main ^{1}H-^{13}C HMBC correlations identified for bicyclomycin produced by SBW25-pJH-BCMclp-PA. Atom numbering relates to Table S3 and is identical to compound numbering in Kohn *et al*. (3).

Position	δ_H, mult. (J in Hz)	δ_C
**1**	-	87.8, C
**3**	3.60, dd, 12.6, 8.5	63.3, CH_2
	3.79, dd, 12.6, 6.2	
**4**	2.48, ddd, 14.3, 8.5, 6.2	35.3, CH_2
**5**	-	149.1, C
**5a**	5.04, br s; 5.35, d, 1.8	115.2, CH_2
**6-OH**	6.88, s	-
**6**	-	81.5, C
**7**	-	169.6, C
**8-NH**	8.72, s	-
**9**	-	166.3, C
**10-NH**	8.96, s	-
**1'**	3.89, d, 7.1	70.3, C
**1'-OH**	5.32, d, 7.5	-
**2'**	-	77.1, C
**2'-CH_3**	1.16, s	23.9, CH_3
**2'-OH**	4.53, s	-
**3'**	1.29, m	66.3, CH_2
**3'-OH**	5.27, br s	-

Table S4. ^{1}H and ^{13}C NMR data for bicyclomycin produced by SBW25-pJH-BCMclp-PA in CD_3SOCD_3.

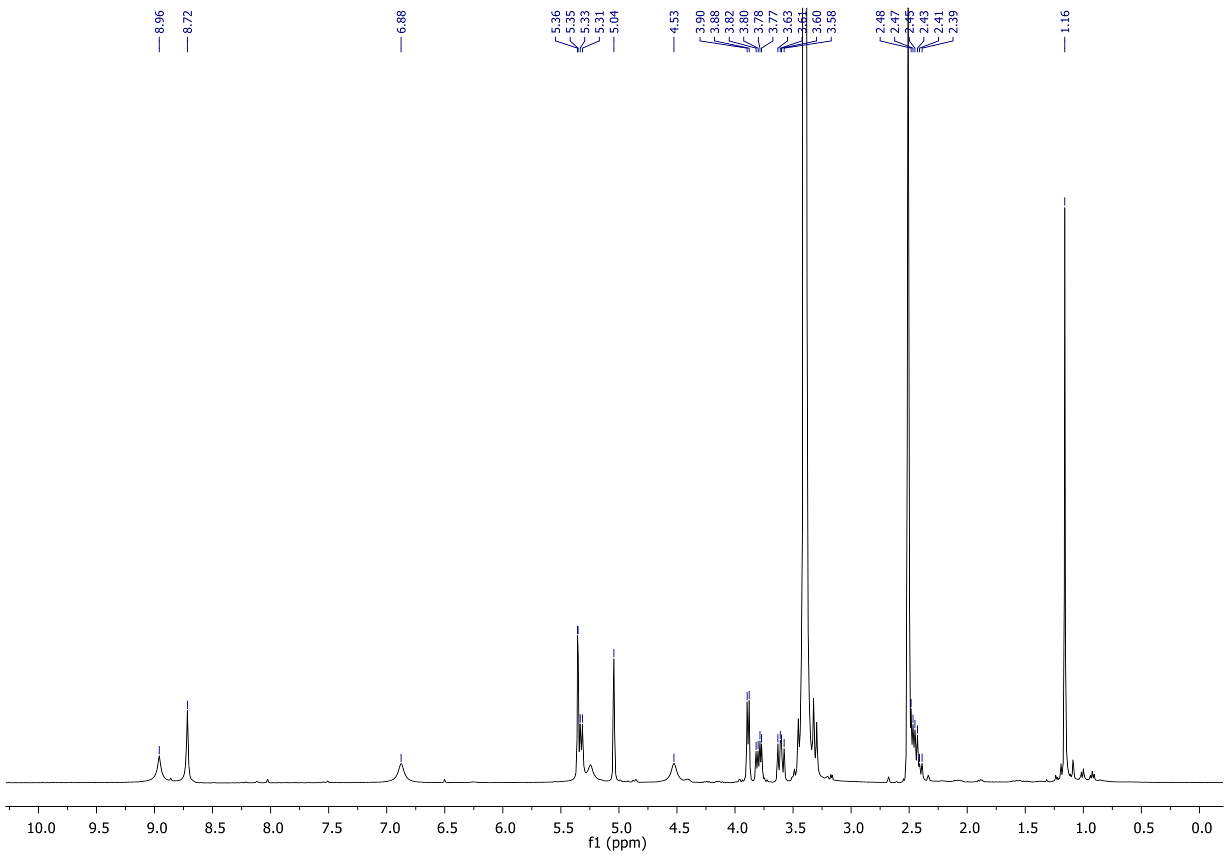

Figure S6. 400 MHz ^{1}H NMR spectrum of bicyclomycin produced by SBW25-pJH-BCMclp-PA in CD_3SOCD_3.

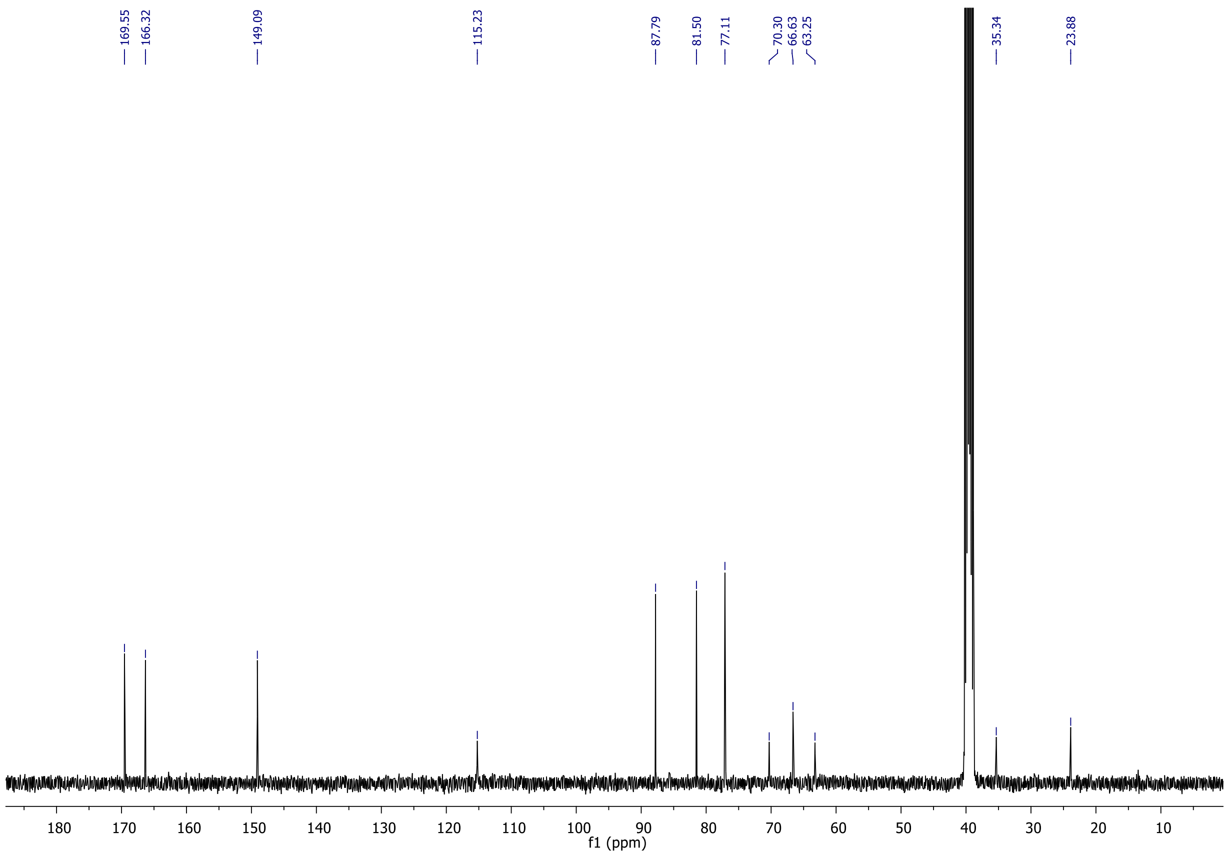

Figure S7. 100 MHz ^{13}C NMR spectrum of bicyclomycin produced by SBW25-pJH-BCMclp-PA in CD_3SOCD_3.

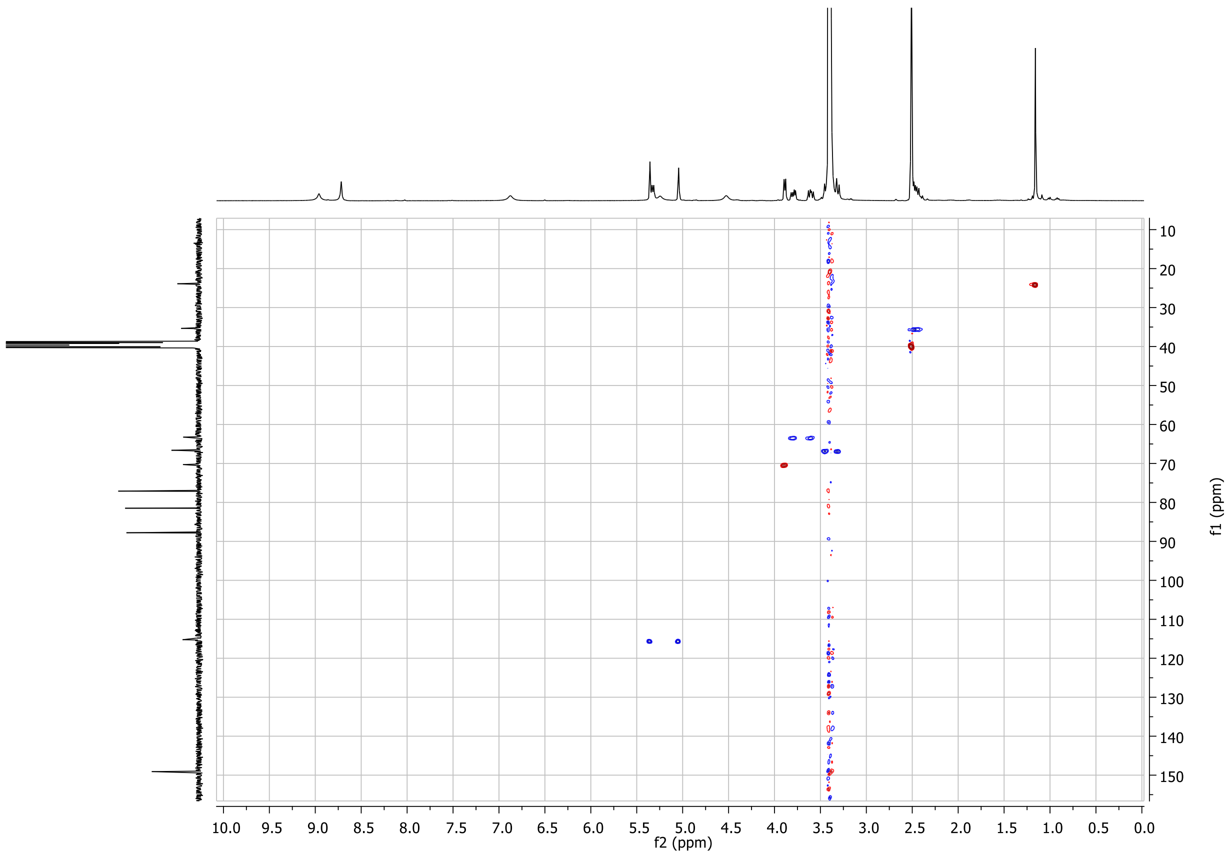

Figure S8. 400 MHz ^{1}H-^{13}C HSQC NMR spectrum of bicyclomycin produced by SBW25-pJH-BCMclp-PA in CD_3SOCD_3.

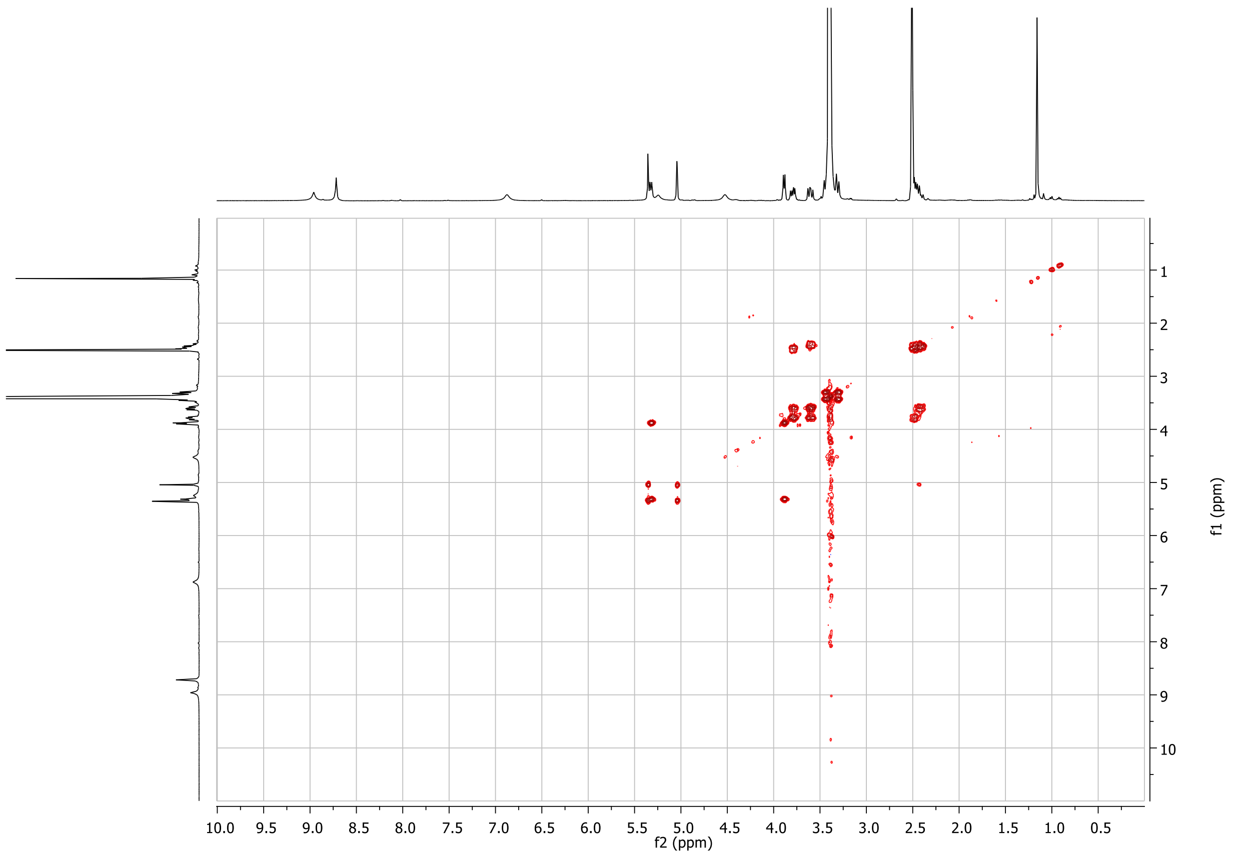

Figure S9. 400 MHz ^{1}H-^{1}H COSY NMR spectrum of bicyclomycin produced by SBW25-pJH-BCMclp-PA in CD_3SOCD_3.

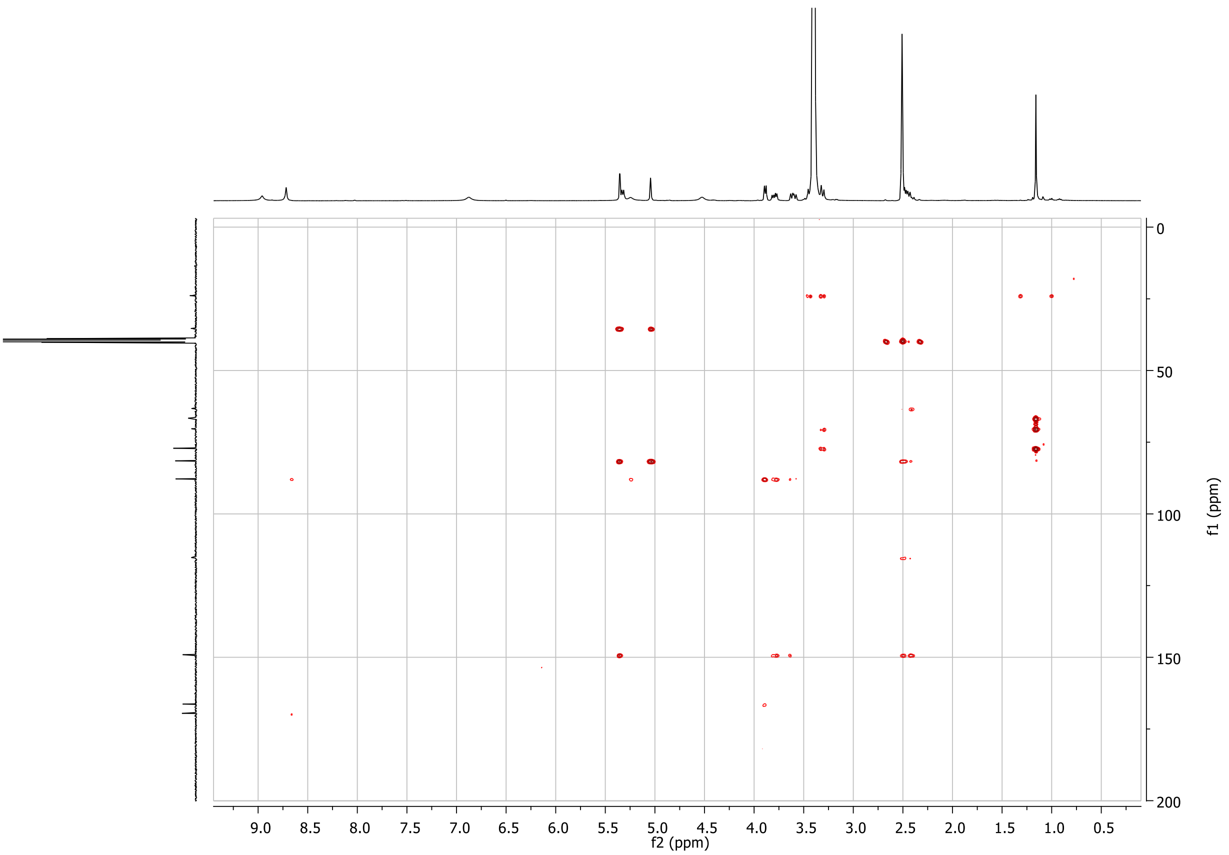

Figure S10. 400 MHz ^{1}H-^{13}C HMBC NMR spectrum of bicyclomycin produced by SBW25-pJH-BCMclp-PA in CD_3SOCD_3.

Strain	Locus tag	Mobile genetic element	Conserved domain
***M. chelonae* CCUG 47445**	BB28_RS01095<br>BB28_RS01100	tRNA-Ser<br>tRNA-Arg	
***M. chelonae* D16R20**	B4407_RS03550<br>B4407_RS03545	tRNA-Ser<br>tRNA-Arg	
***M. chelonae* D16R2**	B4391_RS18230<br>B4391_RS18235	tRNA-Ser<br>tRNA-Arg	
***M. chelonae* 15513**	BKG80_RS00985<br>BKG80_RS00990	tRNA-Ser<br>tRNA-Arg	
***M. chelonae* 15514**	BKG81_RS06600<br>BKG81_RS06660<br>BKG81_RS06665<br>BKG81_RS06670<br>BKG81_RS06675	Resolvase<br>Site-specific phage integrase<br>tRNA-Phe<br>tRNA-Asp<br>tRNA-Glu	pfam00239<br>pfam00589
***W. herbipolensis* ARP1**	TU34_RS19365	IS110 family transposase	pfam02371/pfam01548
***S. hygroscopicus* XM201**	---	---	---
***S. violaceusniger* NRRL F-8817**	ADL28_RS46050<br>ADL28_RS46040<br>ADL28_RS16000	Transposase DDE_Tnp_1_4 domain-containing protein<br>Tn3 transposase DDE domain-containing protein<br>Tn3 transposase DDE domain-containing protein	pfam13701<br>Pfam01526<br>Pfam01526
***S. castelarensis* NRRL B-24289**	BZY55_RS12845<br>BZY55_RS12840<br>BZY55_RS12795	Serine recombinase (resolvase domain)<br>Transposase DDE_Tnp_1_4 domain-containing protein<br>Tn3 transposase DDE domain- containing protein	cd03768/ pfam00239<br>pfam13701<br>pfam01526
***S. kanamyceticus* NRRL B-2535**	---	---	---
***S. formicae* KY5**	---	---	---
***S. cinnamoneus* DSM 41675**	---	---	---
***S. platensis* DSM 40041**	BG653_RS05930	Transposase DDE_Tnp_4 superfamily endonuclease	pfam13359
***S. ossamyceticus* NRRL B-3822**	---	---	---
***A. spheciospongae* EG49**	UO65_RS00270<br>UO65_RS00275	Integrase<br>Transposase DDE domain-containing protein	pfam00665/ pfam13683<br>pfam01609
***T. mobilis* MCCC 1A02139**	AUP44_RS28005	Putative IS4/5 family transposase	pfam13340
***B. plantarii* ATCC 43733**	bpln_RS33885<br>bpln_RS33900<br>bpln_RS33905<br>bpln_RS10520	Transposase DDE_Tnp_1_5 superfamily endonuclease<br>Putative IS4/5 family transposase<br>Transposase<br>IS66 Orf2 like protein	pfam13737<br>pfam13340<br>pfam01527<br>pfam05717
***P. aeruginosa* SCV20265**	---	---	---

Table S5. Mobile genetic elements and tRNA genes surrounding the *bcm* cluster in different bacteria.

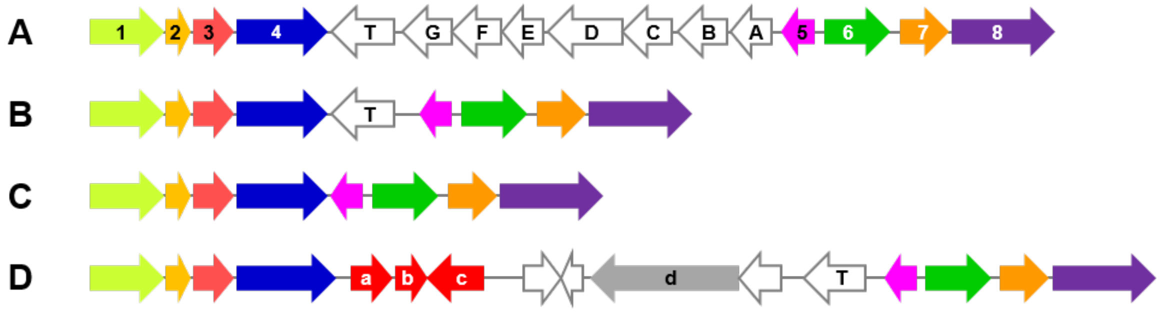

1 – *glmU*
2 – M23 peptidase
3 – *glmR*
4 – *glmS*

5 – HAD-IB hydrolase
6 – Class I methyltransferase
7 – TRAP transporter permease
8 – TRAP transporter solute binding protein

a – Tn7 transposase TnsA protein
b – Tn7 transposase TnsB protein
c – Transposase
d – Superfamily II helicase

Figure S11. Genomic context of the *bcm* cluster (genes A to T) in *P. aeruginosa* and synteny of its flanking genes. (A) Schematic representation of the genes surrounding the *bcm* cluster in all *bcm*-positive *P. aeruginosa* strains, including SCV20265, M18 and ATCC 14886. (B) Organization of the flanking genes in *bcm*-minus strains that feature a *bcmT* homolog (99% identity, 100% coverage) adjacent to *glmS*, including *P. aeruginosa* PAO1 (AE004091), PA14 (ASWV01000021), ATCC 700888 (AKZF01000541) and LESB58 (FM209186). (C) Example of genetic organization where the flanking genes are contiguous to each other, observed in *P. aeruginosa* PA7 (CP000744) and VRFPA01 (AOBK01000101). (D) Genetic organization in *P. aeruginosa* BL08 (NZ_KI518902), where elements of transposon Tn7 along with a *bcmT* homolog are present instead of the full *bcm* cluster. *bcm* flanking genes are identified by numbers while insertion genes are labelled with lowercase letters. Products encoded by these genes are listed in the legend.

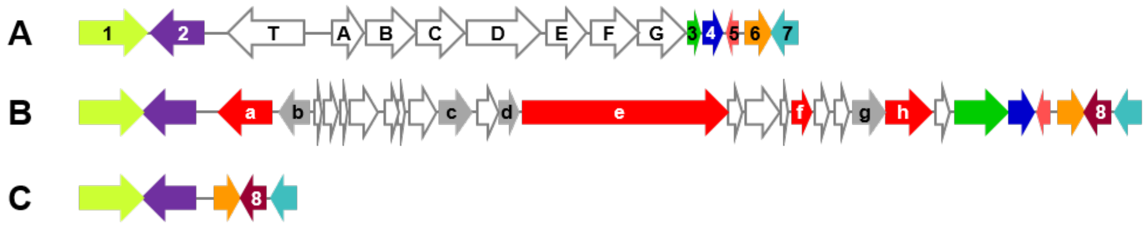

1 – TQXA domain-containing protein
2 – Histidinol-phosphate transaminase
3 – HMGL-like protein
4 – Hypothetical protein
5 – Hypothetical protein
6 – SRPBCC domain-containing protein
7 – TetR transcriptional regulator
8 – N-acetyltransferase

a – Integrase
b – DUF1537 protein
c – HTH domain-containing protein
d – HTH domain-containing protein
e – Phage tail tape measure protein TP901 family
f – HNH endonuclease
g – SAM-dependent methyltransferase
h – uncharacterized phage associated protein

Figure S12. Genomic context of the *bcm* cluster (genes T to G) in *Mycobacterium* and synteny of its flanking genes. (A) Schematic representation of the genes surrounding the *bcm* cluster in *M. chelonae* CCUG 47445. (B) Organization of the same genomic area in *M. abscessus* ATCC 19977 (CU458896) and 6G-0125-R (AKUE01000005) where the *bcm* genes are substituted by a cluster of uncharacterized and phage-associated genes. (C) Gene organization observed in several isolates of *M. abscessus* subsp. *bolletii* (including accessions AKUO01000002, AKUL01000005 and AGQU01000004), where the *bcm* cluster and some of its flanking genes are absent, and no phage is integrated. *bcm* flanking genes are identified by numbers while insertion genes are labelled with lowercase letters. Products encoded by these genes are listed in the legend.

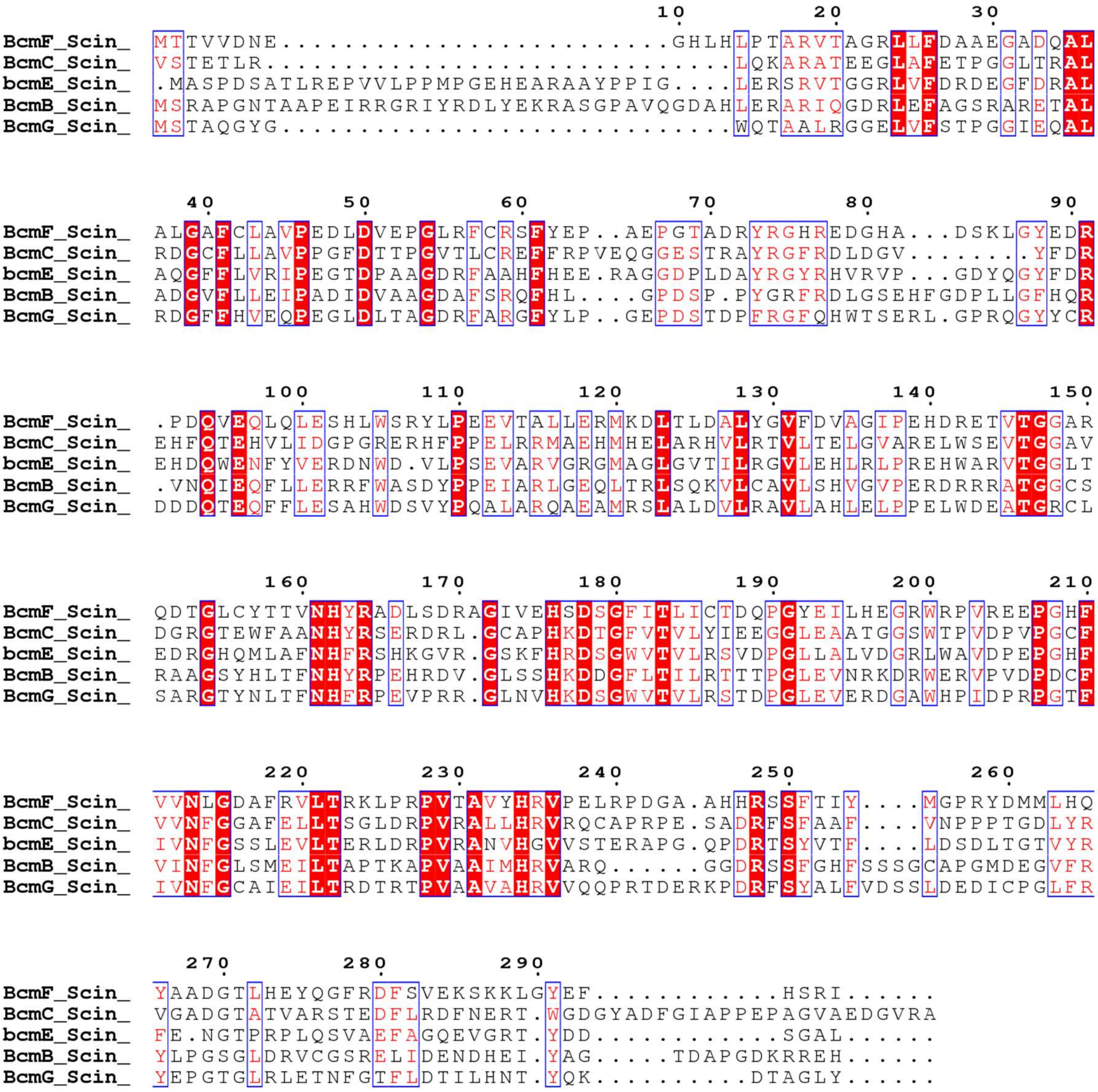

	BcmF_Scin	BcmC_Scin	BcmE_Scin	BcmB_Scin	BcmG_Scin
**BcmF_Scin**	100	33.1	34.2	34.3	32.4
**BcmC_Scin**	33.1	100	37.8	31.1	38.7
**BcmE_Scin**	34.2	37.8	100	31.9	42.0
**BcmB_Scin**	34.3	31.1	31.9	100	42.1
**BcmG_Scin**	32.4	38.7	42.0	42.1	100

Figure S13. Alignment and percentage identity matrix of the *bcm* 2-OG/Fe-dioxygenases from *S. cinnamoneus.* Figure generated using Espript 3.0 (2).

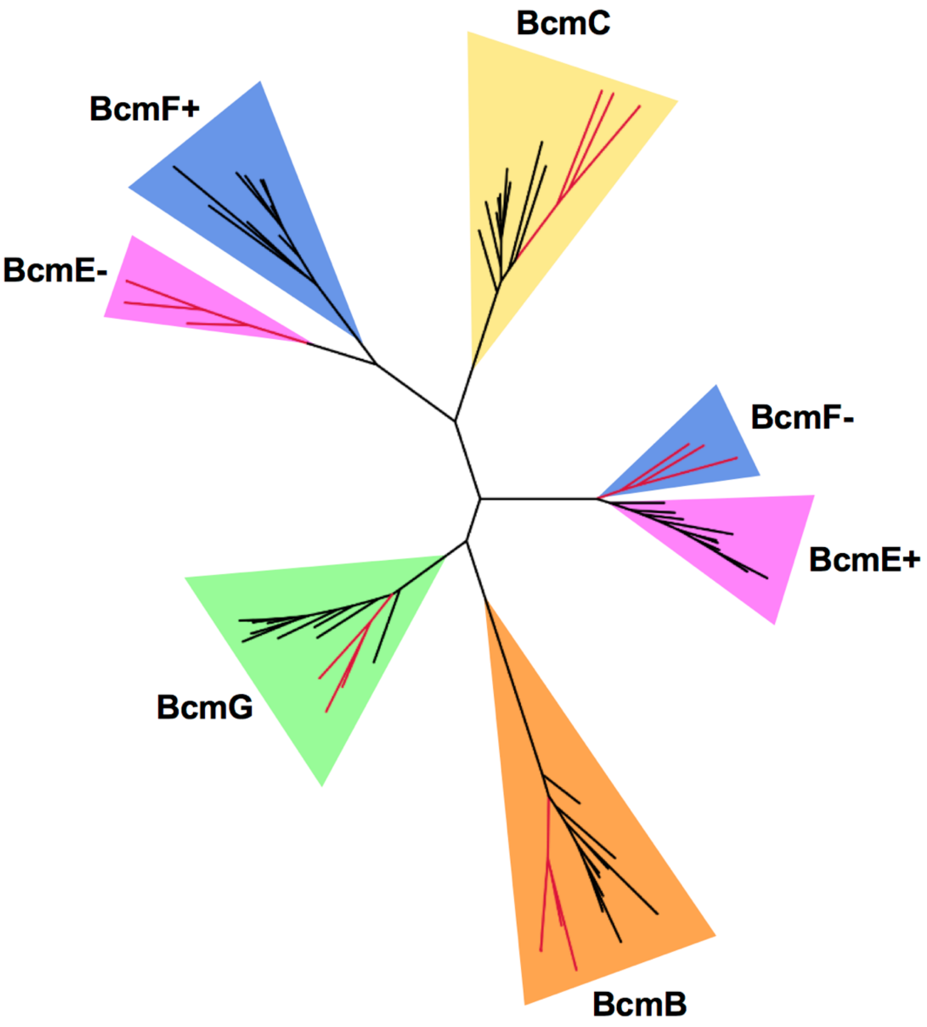

Figure S14. Unrooted version of the phylogenetic tree shown in Fig. 6 without the outgroup. Clade and branch colors are the same as those in Fig. 6.

SUPPLEMENTARY REFERENCES

1. Jacques IB, Moutiez M, Witwinowski J, Darbon E, Martel C, Seguin J, Favry E, Thai R, Lecoq A, Dubois S, Pernodet J-L, Gondry M, Belin P. 2015. Analysis of 51 cyclodipeptide synthases reveals the basis for substrate specificity. Nat Chem Biol 11:721–727.

2. Robert X, Gouet P. 2014. Deciphering key features in protein structures with the new ENDscript server. Nucleic Acids Res 42:W320–W324.

3. Kohn H, Abuzar S, Korp JD, Zektzer AS, Martin GE. 1988. Structural studies of bicyclomycin. J Heterocyclic Chem 25:1511–1517.
